# NlpD links cell wall remodeling and outer membrane invagination during cytokinesis in *Escherichia coli*

**DOI:** 10.1371/journal.pgen.1006888

**Published:** 2017-07-14

**Authors:** Mary-Jane Tsang, Anastasiya A. Yakhnina, Thomas G. Bernhardt

**Affiliations:** Department of Microbiology and Immunobiology, Harvard Medical School, Boston, Massachusetts, United States of America; Max Planck Institute for Terrestrial Microbiology, GERMANY

## Abstract

Cytokinesis in gram-negative bacteria requires the constriction of all three cell envelope layers: the inner membrane (IM), the peptidoglycan (PG) cell wall and the outer membrane (OM). In order to avoid potentially lethal breaches in cell integrity, this dramatic reshaping of the cell surface requires tight coordination of the different envelope remodeling activities of the cytokinetic ring. However, the mechanisms responsible for this coordination remain poorly defined. One of the few characterized regulatory points in the envelope remodeling process is the activation of cell wall hydrolytic enzymes called amidases. These enzymes split cell wall material shared by developing daughter cells to facilitate their eventual separation. In *Escherichia coli*, amidase activity requires stimulation by one of two partially redundant activators: EnvC, which is associated with the IM, and NlpD, a lipoprotein anchored in the OM. Here, we investigate the regulation of amidase activation by NlpD. Structure-function analysis revealed that the OM localization of NlpD is critical for regulating its amidase activation activity. To identify additional factors involved in the NlpD cell separation pathway, we also developed a genetic screen using a flow cytometry-based enrichment procedure. This strategy allowed us to isolate mutants that form long chains of unseparated cells specifically when the redundant EnvC pathway is inactivated. The screen implicated the Tol-Pal system and YraP in NlpD activation. The Tol-Pal system is thought to promote OM invagination at the division site. YraP is a conserved protein of unknown function that we have identified as a new OM-localized component of the cytokinetic ring. Overall, our results support a model in which OM and PG remodeling events at the division site are coordinated in part through the coupling of NlpD activation with OM invagination.

## Author summary

Gram-negative bacterial cells are surrounded by a complex cell envelope consisting of two membranes, the inner and outer membranes, with a layer of cell wall matrix sandwiched between them. Antibiotics have a difficult time penetrating this envelope such that it represents a major drug-resistance determinant of this class of bacteria. An understanding of the basic mechanisms required for envelope assembly therefore promises to teach us how to defeat this barrier and make gram-negative infections easier to treat. Here, we investigate the regulation of cell wall remodeling at the division site of the model gram-negative bacterium *Escherichia coli*. We find that one of the major factors activating cell wall remodeling called NlpD must be localized to the outer membrane to properly control its activity. Furthermore, our genetic analysis implicates a protein complex involved in outer membrane invagination during cytokinesis in promoting NlpD activity. Our results thus identify NlpD as an important mediator connecting cell wall remodeling and outer membrane invagination at the division site of gram-negative bacteria and highlight it as a potential target for agents that disrupt the envelope.

## Introduction

Gram-negative bacterial cells are surrounded by a complex envelope consisting of an inner (cytoplasmic) membrane (IM), a polysaccharide matrix called the peptidoglycan (PG) cell wall, and an outer membrane (OM) [1]. This triple-layered surface structure is essential for cell integrity and serves as a formidable barrier hindering the penetration of drug molecules into cells. A greater understanding of envelope assembly and remodeling therefore promises to identify better ways of getting drugs into this class of bacteria for the development of effective anti-gram-negative therapeutics. In this regard, cell division is an attractive area of study because the process involves a substantial remodeling of the envelope where all three layers are constricted in concert. This transformation is carried out by a ring-shaped cytokinetic apparatus known as the divisome, which is organized by polymers of the tubulin-like FtsZ protein and contains dozens of proteins important for cell division [2]. Although most of the components of the divisome have been identified, relatively little is known about how it orchestrates envelope constriction to ensure that cell wall synthesis and remodeling are coordinated with the invagination of the two membrane layers.

During division, the divisome synthesizes new cell wall material composed of alternating β-1,4-linked N-acetyl-glucosamine (GlcNAc) and N-acetyl-muramic acid (MurNAc) sugar polymers crosslinked via amide bonds between stem peptides attached to MurNAc. The PG produced by the divisome, called septal PG, is initially shared by the developing daughter cells [2]. To shape the daughter cell poles and promote their separation, the septal PG must be carefully split by enzymes that cleave bonds in the PG matrix. In *Escherichia coli*, septal PG cleavage is largely mediated by the periplasmic LytC-type N-acetylmuramyl-L-alanine amidases (AmiA, AmiB, and AmiC) [3,4], which cleave the linkages attaching the stem peptides to the glycan strands. These enzymes are autoinhibited and require activation by the divisome proteins EnvC and NlpD [5,6]. Each activator possesses a degenerate LytM (dLytM) domain, which is related to metallo-endopeptidases that cleave PG but is missing residues needed for the coordination of the catalytic Zn^2+^ ion [6,7]. Accordingly, the crystal structure of the dLytM domain of EnvC lacks a Zn^2+^ ion [8]. Instead, mutational analysis suggests that EnvC uses this degenerate active site cleft to stimulate the release of amidase autoinhibition and promote septal PG hydrolysis. The dLytM domain of NlpD most likely functions similarly, but its function has not been investigated in detail.

The amidases and their regulators are organized into two partially redundant activation pathways. EnvC specifically stimulates AmiA and AmiB *in vitro*, whereas NlpD activates AmiC [6]. Inactivation of the individual activators or amidases does not lead to a significant cell separation defect. A severe phenotype is only observed upon deletion of multiple amidase genes or by blocking the activity of both dLytM activators [3,4,7]. When both the EnvC and NlpD pathways are inactivated, cells propagate as long chains with cell units linked by a shared septal PG layer and a partially constricted outer membrane layer [7]. Notably, the barrier function of the outer membrane in these chains is compromised, rendering them hypersensitive to detergents, antibiotics, and cationic antimicrobial peptides [9–12].

Tight control over amidase activation is essential to prevent the formation of breaches in the cell wall structure that can result in cell lysis [5]. The regulation of amidase activation by EnvC is relatively well-understood. The activator is anchored to the outer face of the IM and recruited to division sites via a direct interaction between its coiled-coil (CC) domain and external loop domains of the ATP-binding cassette (ABC) transporter-like complex FtsEX [13]. In addition to its function in protein localization, the CC domain also seems to be involved in EnvC regulation [6]. In support of this possibility, structural studies of the related cell separation protein PcsB from *Streptococcus pneumoniae* indicate that its CC domain embraces the PG hydrolase-like domain in what would be predicted to be an auto-inhibited conformation [14]. Variants of FtsEX lacking ATPase activity still recruit EnvC to the division site but fail to promote septal PG splitting [6,13]. This observation has led to the proposal that amidase activation by EnvC is stimulated by conformational changes in the FtsEX complex as it undergoes an ATPase cycle [13]. Similar connections between FtsEX and PG hydrolase activation in other organisms suggest that this may be a widespread mechanism for regulating cell wall cleavage in bacteria [15–17]. Recent results also suggest a role for FtsEX in controlling PG synthesis by the divisome, thereby linking the processes of PG synthesis and hydrolysis during division [18].

Here, we investigate the control of AmiC activation by the lipoprotein NlpD, which in contrast to EnvC, has remained poorly characterized. Structure-function analysis revealed that OM localization of NlpD is critical for proper control of its amidase activation activity. Variants that mislocalize to the periplasm caused an elevated frequency of cell lysis due to spurious AmiC activation. To identify additional factors involved in the NlpD cell separation pathway, we also developed a genetic screen using a flow cytometry-based enrichment procedure. This strategy allowed us to isolate mutants that form long chains of unseparated cells specifically when the redundant EnvC pathway is inactivated. The screen implicated the Tol-Pal system and YraP in NlpD activation. The Tol-Pal system is thought to promote OM invagination at the division site. YraP is a conserved protein of unknown function that we have identified as a new OM-localized component of the cytokinetic ring. Overall, our results support a model in which OM and PG remodeling events at the division site are coordinated in part by the coupling of NlpD activation with OM invagination.

## Results

### Domain organization of NlpD

**Fig 1:**
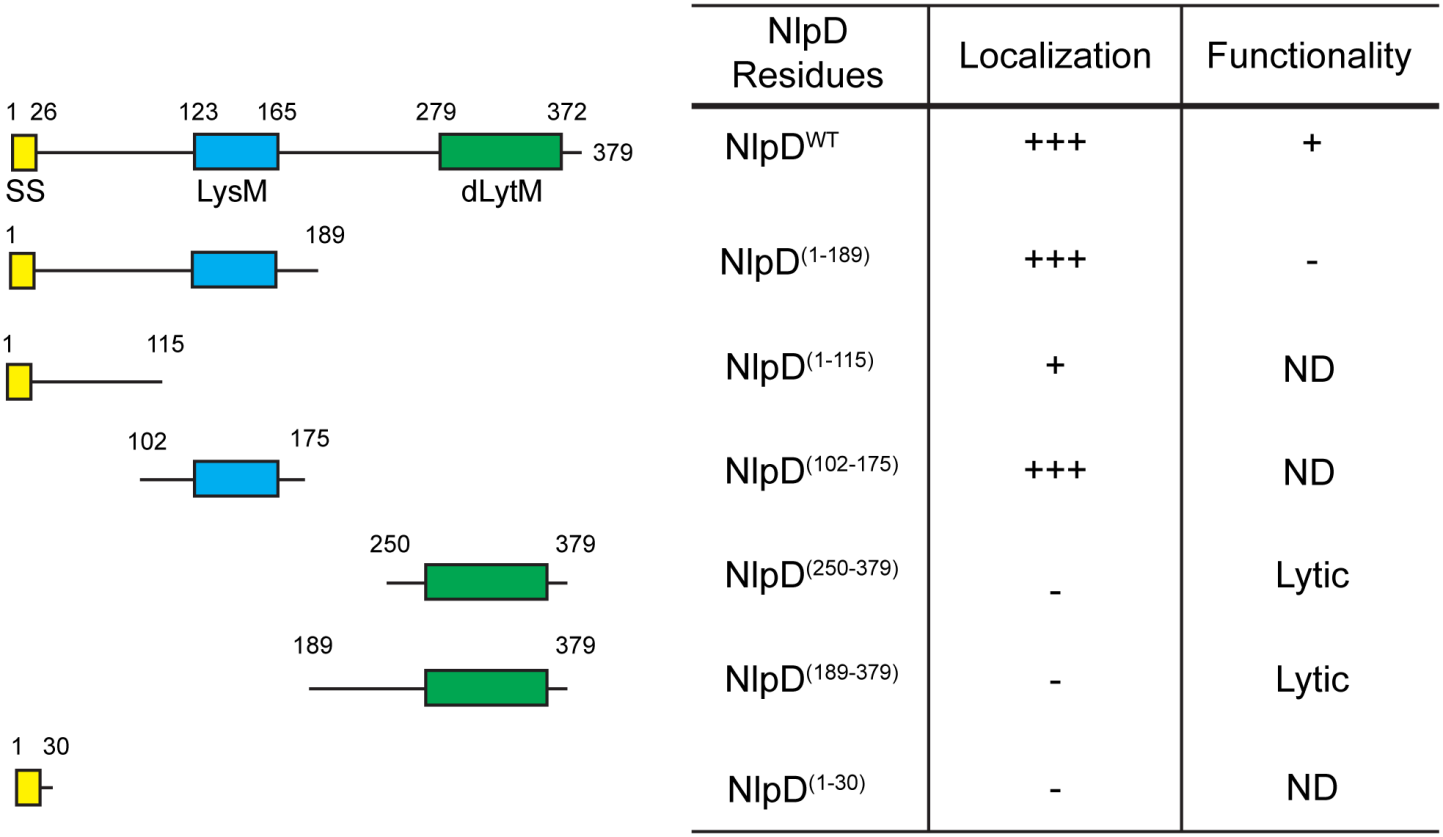
Structure-function analysis of NlpD. The domain organization of NlpD is illustrated. Indicated are the signal sequence (SS; yellow), lysin motif (LysM; blue), and the degenerate LytM domain (dLytM; green). Also shown are the NlpD truncations that were expressed under the control of the IPTG-inducible lactose promoter either as an untagged protein or as a C-terminal mCherry fusion. Truncations lacking ^SS^NlpD are expressed as soluble periplasmic proteins fused to the DsbA signal peptide. Columns indicate (i) the NlpD residues present in each truncation, (ii) whether the fusion to mCherry accumulated at division sites strongly (+++), poorly (+), or appeared evenly distributed along the periphery of the cell (-), and (iii) whether the untagged truncation could (+) or could not (-) compensate for the loss of endogenous NlpD for proper cell separation. ND, not determined.

NlpD is an outer membrane lipoprotein consisting of multiple domains: a lysin motif (LysM) domain [19–21], a dLytM domain [8], and two linker regions, one at the N-terminus and another between the LysM and dLytM domains (Fig 1). The linker regions are predicted to be disordered/unstructured by DISOPRED [22]. The LysM domain is a common motif in envelope proteins and is associated with PG binding activity [20,21]. The dLytM domain has been reported to mediate amidase activation by the other dLytM factor EnvC [6,8]. To begin dissecting the regulation of amidase activation by NlpD, we initiated a structure-function analysis of the protein in order to determine which domains are necessary and/or sufficient for recruitment to the divisome and amidase regulation. The results are summarized in Fig 1.

### The LysM domain of NlpD is necessary and sufficient for septal localization

In order to determine which region of NlpD is the localization determinant, we constructed different NlpD variants with the fluorescent protein mCherry fused at the C-terminus (Fig 1). We expressed truncated versions of NlpD lacking either the dLytM domain or both the dLytM and LysM domains. Additionally, we also expressed soluble periplasmic variants containing only the dLytM domain (NlpD^dLytM^) or only the LysM domain (NlpD^LysM^). These domains were fused to the signal sequence of DsbA for Sec-mediated export into the periplasm [23]. Finally, we expressed mCherry fused to the signal sequence of NlpD plus 4 additional amino acids after the acylated cysteine to assess the effects of lipidation and OM localization on localization pattern. All the constructs were expressed under the control of the IPTG-inducible lactose promoter (P_*lac*_) from a chromosomally integrated plasmid in an *nlpD* null strain. Additionally, all fusion proteins were found to be largely intact by western blot analysis using an antibody against the mCherry portion of the fusion (S1A Fig).

Because the *nlpD* gene contains a promoter for the downstream *rpoS* gene, we did not use a complete *nlpD* deletion as our null allele. RpoS is an alternative sigma factor and global regulator that controls the expression of a large number of genes in response to a variety of stresses [24]. A complete deletion of *nlpD* results in defects in *rpoS* expression [25]. In order to inactivate NlpD with minimal effects on *rpoS* expression, we constructed an in-frame deletion of the coding sequence for the N-terminal lipoprotein signal sequence of NlpD (^*ΔSS*^*nlpD*) to prevent its export to the periplasm where it normally functions. Importantly, ^*ΔSS*^*nlpD* and *ΔnlpD* mutants displayed the same severe chaining phenotype when combined with a *ΔenvC* mutation, suggesting that a ^*ΔSS*^*nlpD* strain behaves like an *nlpD* null strain with respect to amidase activation. We therefore use ^*ΔSS*^*nlpD* as our null allele throughout this study.

**Fig 2:**
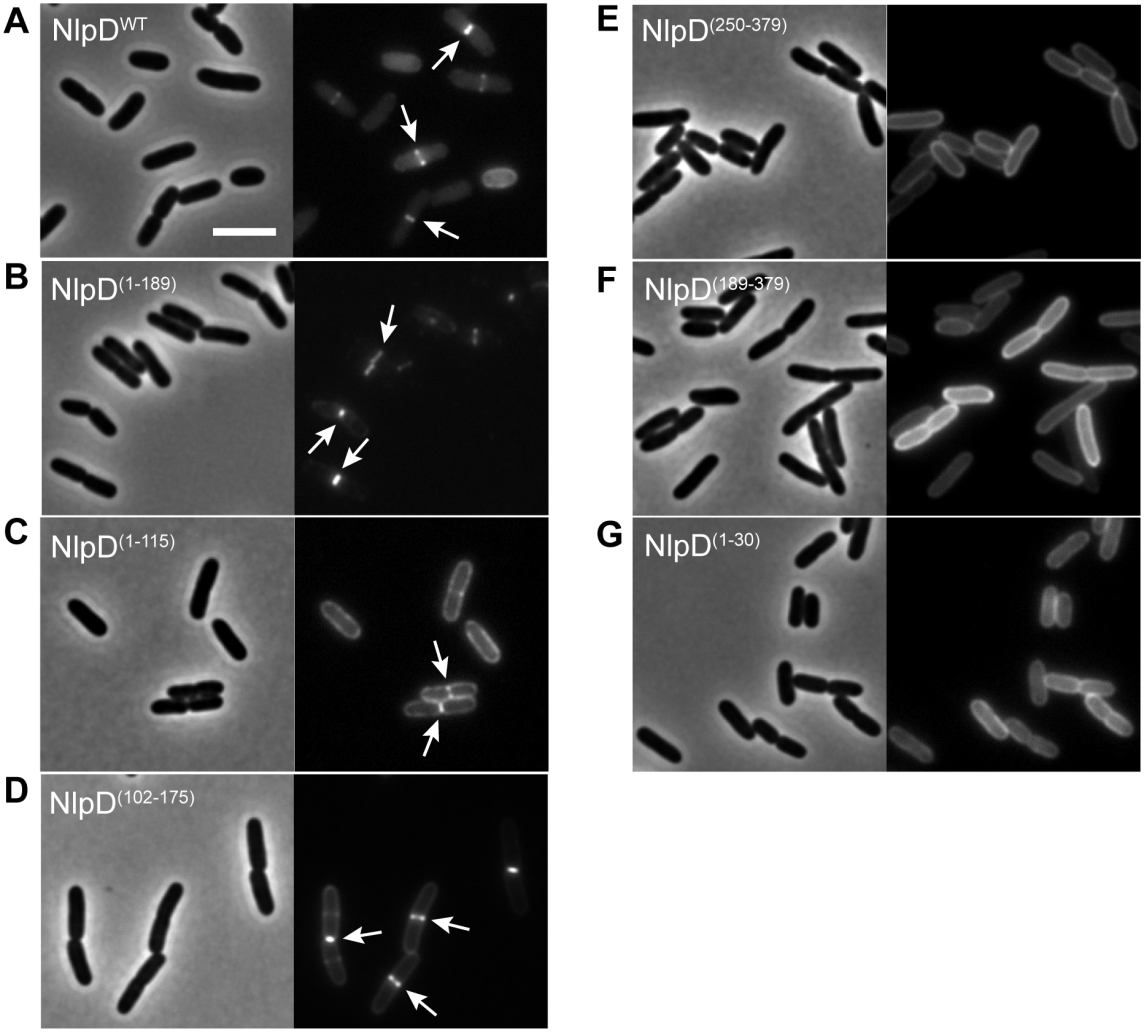
Localization of NlpD fusions in ^*ΔSS*^*nlpD* cells. Overnight cultures of MT47 (^*ΔSS*^*nlpD*) harboring the integrated expression constructs (**A**) attHKNP20 (*P*_*lac*_::*nlpD*^*WT*^*-mCherry*), (**B**) attHKMT101 (*P*_*lac*_::*nlpD*^*(1–189)*^*-mCherry*), (**C**) attHKMT103 (*P*_*lac*_::*nlpD*^*(1–115)*^*-mCherry*), (**D**) attHKMT178 (*P*_*lac*_::^*ss*^*dsbA-nlpD*^*(102–175)*^*-mCherry*), (**E**) attHKMT180 (*Pl*_*ac*_::^*ss*^*dsbA-nlpD*^*(250–379)*^*-mCherry*), (**F**) attHKMT182 (*P*_*lac*_::^*ss*^*dsbA-nlpD*^*(189–379)*^*-mCherry*), or (**G**) attHKMT149 (*P*_*lac*_::*nlpD*^*(1–30)*^*-mCherry*) were diluted in minimal M9-maltose medium supplemented with 25μM (**E-F**), 50μM (**A-B**), 100μM (**C-D**), or 150μM (**G**) IPTG. Cells were grown at 30°C to an OD_600_ of 0.15–0.25 before they were visualized on 2% agarose pads by phase contrast and fluorescence microscopy. Arrows indicate localization of the protein fusion to division sites. Bar = 4μm.

As expected from previous results [7], the full-length NlpD-mCherry fusion was functional for cell separation and localized to midcell in both constricted and unconstricted cells (Figs 1 and 2A). All constructs containing the LysM domain, whether lipidated or free-floating, also displayed midcell localization (Figs 1, 2B and 2D), indicating that the LysM domain is sufficient for localization. A fusion with just the N-terminal linker also appeared to have some affinity for the midcell, although the enrichment at the division site was much reduced compared to wild-type NlpD (Figs 1 and 2C). This localization was not due to lipidation since a fusion of mCherry to just the NlpD signal sequence did not show any specific midcell localization (Figs 1 and 2G). Finally, the soluble periplasmic NlpD^dLytM^ variants (NlpD^(250–379)^-mCherry and NlpD^(189–379)^-mCherry) were dispersed throughout the periplasm, suggesting that although this domain is responsible for amidase activation, it does not promote NlpD recruitment to the division site (Figs 1, 2E and 2F). Taken together, these results suggest that, similar to other LysM-containing division proteins [26–28], the LysM module of NlpD is both necessary and sufficient for proper localization to the septal ring.

### The dLytM domain of NlpD is required for function and causes lysis when periplasmic

**Fig 3:**
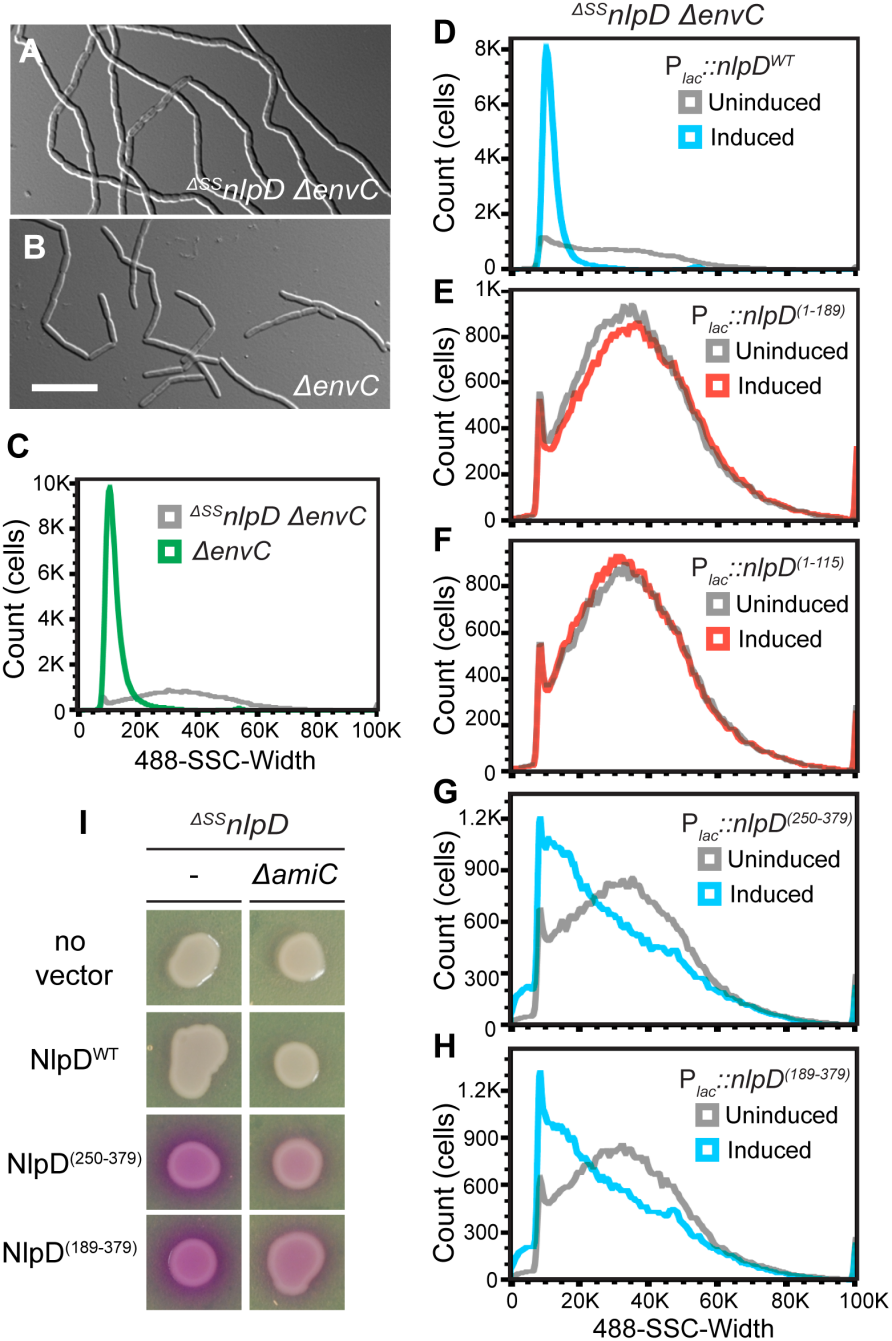
Only full-length NlpD properly promotes cell separation. (**A-C**) MT50 (^*ΔSS*^*nlpD ΔenvC*) or TB140 (*ΔenvC*) cells were grown in minimal M9-maltose medium at 37°C to an OD_600_ of 0.2–0.3 before visualization on 2% agarose pads with DIC optics (**A-B**) or analyzed by flow cytometry (**C**). Bar = 10μm. (**D-H**) Overnight cultures of MT50 harboring the integrated expression constructs (**D**) attHKMT20 (*P*_*lac*_::*nlpD*^*WT*^), (**E**) attHKMT102 (*P*_*lac*_::*nlpD*^*(1–189)*^), (**F**) attHKMT104 (*P*_*lac*_::*nlpD*^*(1–115)*^), (**G**) attHKMT179 (*Pl*_*ac*_::^*ss*^*dsbA-nlpD*^*(250–379)*^), or (**H**) attHKMT181 (*P*_*lac*_::^*ss*^*dsbA-nlpD*^*(189–379)*^) were diluted in minimal M9-maltose medium and grown at 37°C. Mid-log cultures were then backdiluted into M9-maltose medium with or without the inducer IPTG. Cells were further grown at 37°C to an OD_600_ of 0.2–0.3 before flow cytometry analysis. Histograms of cultures that were either uninduced (grey) or induced with 150μM (**D, G-H**, blue) or 1mM (**E-F**, red) IPTG were overlayed. (**I**) Cells of MT122 (^*ΔSS*^*nlpD*) and MT123 (^*ΔSS*^*nlpD ΔamiC*) alone or harboring the integrated constructs attHKMT20 (*P*_*lac*_::*nlpD*^*WT*^), attHKMT179 (*Pl*_*ac*_::^*ss*^*dsbA-nlpD*^*(250–379)*^), or attHKMT181 (*P*_*lac*_::^*ss*^*dsbA-nlpD*^*(189–379)*^) were grown in LB at 30°C. Following normalization for cell density (OD_600_ = 0.5), 5 μl of the resulting cultures was spotted on LB agar containing 150μM IPTG and 20 μg/ml CPRG. The plates were incubated at 30°C and photographed after 14 hours.

The functional importance of the subdomains of NlpD for cell separation was also investigated using the truncations described above, but in this case as untagged proteins in order to avoid potential negative effects on NlpD functionality due to the mCherry tag. To assess their function, the different truncations were each expressed under the control of the P_*lac*_ promoter from a chromosomally integrated plasmid in a strain lacking both NlpD and EnvC (Fig 1). Without a complementing construct, the ^*ΔSS*^*nlpD ΔenvC* strain formed very long chains of cells that failed to separate (Fig 3A). Full correction of the NlpD defect in this strain should restore its division function such that it resembles a single *ΔenvC* strain, which primarily forms long cell pairs and short chains [29] (Fig 3B). It can be difficult to quantitatively compare the severity of cell chaining phenotypes between mutants using microscopy alone because many cell chains extend outside of the field of view. We therefore used flow cytometry to complement our phenotypic analysis. In this assay, cells with increased cell length, such as chaining cells or smooth filaments, cause a significant increase in the detected side scatter pulse width (SSC-W) relative to normally dividing cells. The *ΔenvC* strain, which is relatively uniform in size, showed a tight peak in the SSC-W distribution by flow cytometry (Fig 3C). On the other hand, the long and heterogeneously sized cell chains of the ^*ΔSS*^*nlpD ΔenvC* strain displayed a broad SSC-W distribution. Thus, this assay allowed us to assess on a cell population-level how efficiently the different NlpD truncations rescued the cell separation defect of the ^*ΔSS*^*nlpD ΔenvC* double mutant (Fig 3D–3H).

Expression of wild-type NlpD from the integrated plasmid complemented the ^*ΔSS*^*nlpD* mutation, and converted the broad SSC-W profile of the double mutant to one resembling the tight distribution of a single *ΔenvC* strain (Figs 1, 3D and S2A Fig). Variants lacking the dLytM domain all failed to suppress the cell chaining phenotype (Figs 1, 3E, 3F and S2B and S2C Fig). At least one of these variants (NlpD^(1–189)^) was expressed and remained largely intact as assessed by immunoblot analysis. However, it is possible that the truncated product observed for this variant affects the ability of the NlpD^(1–189)^ to correct the cell chaining phenotype of ^*ΔSS*^*nlpD ΔenvC* cells (S1B Fig). The other variant, NlpD^(1–115)^, was not detected, either due to the lack of robust antigenic epitopes in this truncation or proteolytic degradation, so we could not determine its functionality. Overall, the results indicate that, similar to EnvC, the dLytM domain of NlpD is required for amidase activation and cell separation *in vivo*.

To test whether the dLytM domain of NlpD is sufficient for NlpD activity, the soluble NlpD^dLytM^ variants (NlpD^(250–379)^ and NlpD^(189–379)^) were produced in the ^*ΔSS*^*nlpD ΔenvC* strain. Neither variant suppressed the cell separation defect as observed by microscopy, but they did appear to reduce the total cell length and increase the number of cells with a lower SSC-W when analyzed by flow cytometry (Figs 1, 3G, 3H and S2D and S2E Fig). Because the EnvC dLytM domain is lytic when produced in isolation [6], we suspected that the NlpD^dLytM^ variants may also promote aberrant amidase activation. To test for lytic activity, we used the β-galactosidase substrate chlorophenyl red-β-D-galactopyranoside (CPRG), which fails to penetrate the *E*. *coli* envelope and cannot be processed by cytoplasmic LacZ unless cells lyse and/or become more permeable [30]. Indeed, expression of both NlpD^dLytM^ variants resulted in a positive red signal on CPRG-containing medium (Fig 3I). This phenotype was not caused by NlpD^WT^, and was largely suppressed by inactivation of AmiC, the target amidase of NlpD (Fig 3I). Thus, the lytic activity of the NlpD^dLytM^ variants is most likely the result of spurious AmiC activation, rather than a non-specific defect resulting from the production of truncated proteins in the periplasm. However, some residual lysis is observed even in AmiC-defective cells and is probably caused by promiscuous activation of the other amidases, AmiA and AmiB, by the NlpD^dLytM^ variants as has been observed previously for certain AmiB derivatives [5]. We conclude that although the dLytM domain is critical for NlpD activity and sufficient for amidase activation, it is unable to promote proper cell separation on its own and may require additional domains of the NlpD protein for its proper regulation.

### Outer membrane localization of NlpD is important for proper function and regulation

**Fig 4:**
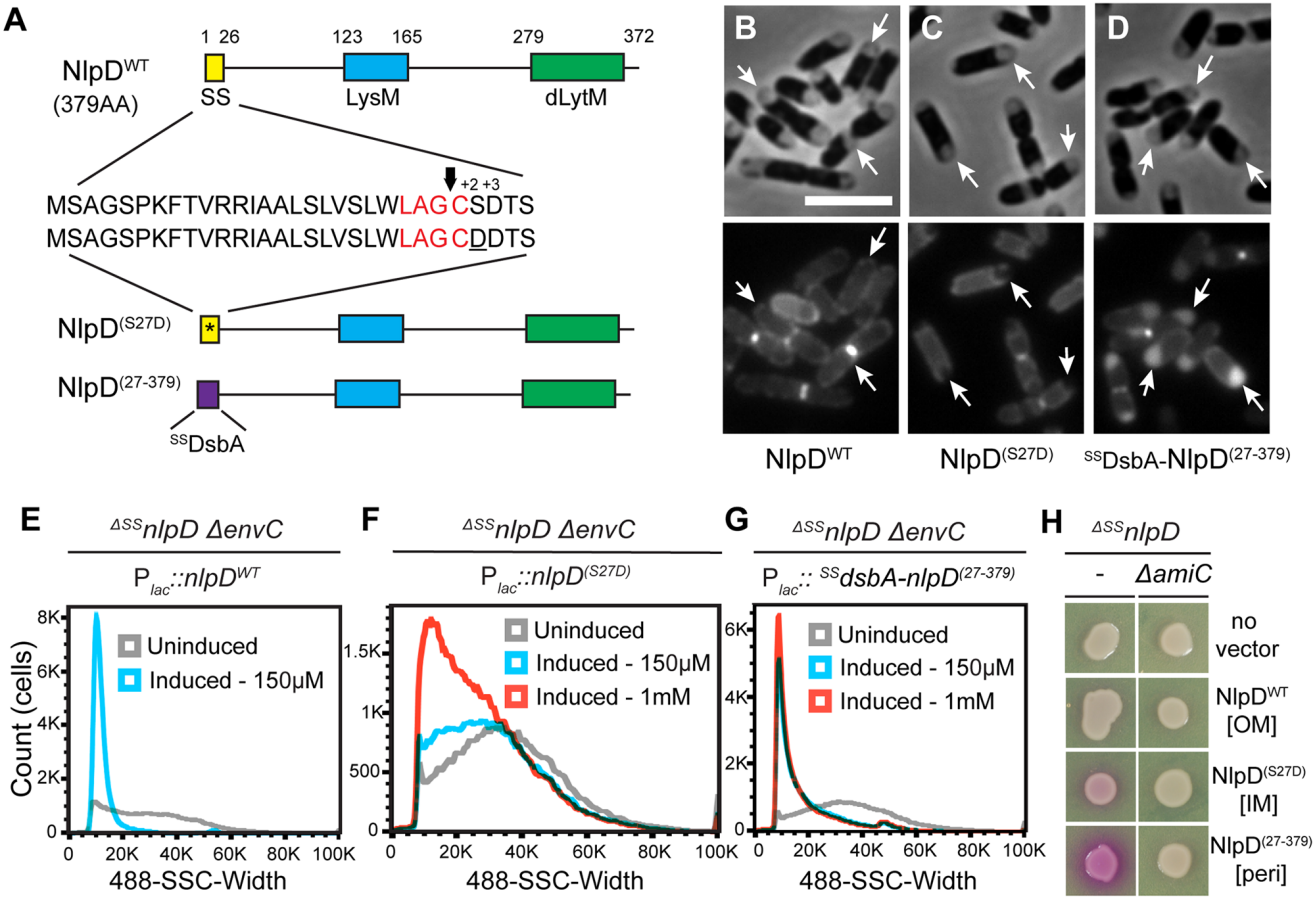
OM localization of NlpD is required for proper cell separation. (**A**) The domain structure of NlpD is illustrated as in Fig 1. Details of the signal sequence are presented with the lipobox in red and the arrow indicating the cleavage site just before the acylated cysteine. The IM-retained variant (NlpD^(S27D)^) contains a mutated signal sequence (indicated by the asterisk) with an aspartate at the +2 position after the acylated cysteine (underlined). The soluble periplasmic variant (NlpD^(27–379)^) is fused to the DsbA signal peptide (purple) that is cleaved upon export to the periplasm via the Sec system. (**B-D**) Cytological assay to determine the subcellular localization of the NlpD variants. Overnight cultures of MT47 (^*ΔSS*^*nlpD*) expressing different NlpD-mCherry fusions from the integrated constructs (**B**) attHKNP20 (*P*_*lac*_::*nlpD*^*WT*^*-mCherry*), (**C**) attHKMT21 (*P*_*lac*_::*nlpD* ^*(S27D)*^*-mCherry*), or (**D**) attHKMT147 (*P*_*lac*_::^*ss*^*dsbA-nlpD* ^*(27–379)*^*-mCherry*) were diluted in minimal M9-maltose medium supplemented with 25 μM (**D**) or 150 μM (**B, C**) IPTG. Cells were grown at 30°C to an OD_600_ of 0.4, washed and then osmotically shocked by resuspension in plasmolysis buffer and the plasmolyzed cells were visualized by phase contrast and fluorescence microscopy. Arrows indicate signals that display a smooth OM peripheral signal in (**B**), track with the inner membrane in (**C**), or fill the increased periplasmic spaces of plasmolysis bays in (**D**). Bar = 4μm. (**E-G**) Overnight cultures of MT50 (^*ΔSS*^*nlpD ΔenvC*) harboring the integrated expression constructs (**E**) attHKMT20 (*P*_*lac*_::*nlpD*^*WT*^), (**F**) attHKMT12 (*P*_*lac*_::*nlpD* ^*(S27D)*^), or (**G**) attHKMT121 (*P*_*lac*_::^*ss*^*dsbA-nlpD* ^*(27–379)*^) were diluted in minimal M9-maltose medium and grown at 37°C until mid-log. Cultures were then backdiluted into M9-maltose medium without (grey histogram) or with 150 μM (blue histogram) or 1 mM (red histogram) IPTG and grown at 37°C to an OD_600_ of 0.2–0.3 before flow cytometry analysis. (**H**) Cells of MT122 (^*ΔSS*^*nlpD*) and MT123 (^*ΔSS*^*nlpD ΔamiC*) alone or harboring the integrated constructs described above were grown and spotted on CPRG agar as described in Fig 3I. The subcellular localization of each NlpD variant is indicated in the square brackets: OM, outer membrane; IM, inner membrane; peri, periplasm.

The behavior of the NlpD^dLytM^ truncations indicated that divisome recruitment and/or OM localization is important for the proper control of AmiC activation by NlpD. To investigate these possibilities further, we generated additional full-length NlpD variants predicted to have an altered localization within the envelope. Firstly, the residue after the acylated cysteine (the +2 position) was altered from a serine to aspartate (NlpD^(S27D)^) to prevent transport of this variant by the Lol system and force its retention in the inner membrane [31] (Fig 4A). We also constructed a variant (^DsbA^NlpD^(27–379)^) in which the first 26 residues, including the lipidated cysteine, were replaced by the signal sequence of DsbA to export a full-length, mature form of NlpD as a soluble periplasmic protein.

In order to assess whether the different variants localized as expected, a cytological assay was used [32]. Cells expressing each variant fused to mCherry at the C-terminus were hyperosmotically shocked to induce plasmolysis and subsequently visualized using both phase contrast and fluorescence microscopy. Upon plasmolysis, the cytoplasm contracted, pulling the inner membrane away from the envelope to form plasmolysis bays (Fig 4B–4D). Wild-type NlpD-mCherry displayed a smooth peripheral fluorescence signal in the plasmolyzed cells, consistent with an OM localization (Fig 4B). On the other hand, the fluorescence pattern of NlpD^(S27D)^-mCherry followed the retracted inner membrane, indicating that this variant is indeed retained in the IM (Fig 4C). Finally, the fluorescent signal of NlpD^(27–379)^-mCherry accumulated in the increased periplasmic space of the plasmolysis bays, which are phase-gray in the phase-contrast images (Fig 4D). Based on these cytological results, we conclude that the different NlpD variants are present in the expected subcellular locations.

Next, we determined whether the NlpD variants with altered localization were functional in amidase activation by assessing their ability to complement the chaining phenotype of ^*ΔSS*^*nlpD ΔenvC* cells. In this case, untagged variants were produced from the P_*lac*_ promoter from a chromosomally integrated plasmid to achieve levels comparable to those produced from natively expressed *nlpD* (S3A Fig). Although all variants were largely intact, a prominent degradation product was present with the periplasmic ^DsbA^NlpD^(27–379)^ variant, and we were concerned about its possible negative effect(s) on the functionality of the full-length protein. To address this concern, we showed that cells lacking *envC* but expressing wild-type endogenous NlpD are able to divide properly in the presence of either the IM-retained NlpD variant (S4B Fig) or the periplasmic ^DsbA^NlpD^(27–379)^ variant (S4C Fig). Hence, neither the NlpD variant nor any degradation product interferes with the ability of wild-type NlpD to activate AmiC and promote cell separation.

As shown above, expression of wild-type NlpD rescued the chaining phenotype of the ^*ΔSS*^*nlpD ΔenvC* double mutant such that cells displayed the mild division defect of a single *ΔenvC* mutant and had a similar flow cytometry profile (Fig 4E and S3B and S3C Fig). Expression of the IM-retained NlpD variant did not suppress the chaining phenotype of the double mutant, even when fully induced, as assessed by both microscopy and flow cytometry (Fig 4F and S3D Fig). Since this variant is stable and intact (S3A Fig) and at least partly able to localize to division sites (S4E Fig), this finding suggests that anchoring NlpD to the IM greatly reduced its ability to activate its cognate amidase AmiC. Notably, the soluble periplasmic NlpD variant also failed to fully complement the *nlpD* null phenotype, even though it displayed proper septal localization (Fig 4G and S4F Fig). Although production of this variant led to a reduction in chain length as assessed by both microscopy and flow cytometry (Fig 4G and S3E Fig), many chaining cells were still observed in the population by microscopy and detected in the flow cytometry histogram as a tailed distribution of SSC-W towards longer cells. Thus, despite retaining the ability to activate AmiC for cell separation, this NlpD variant does so less efficiently than the wild-type protein. Because ^DsbA^NlpD^(27–379)^ induced an AmiC-dependent red signal on CPRG-containing medium (Fig 4H), it is difficult to know how much of the reduced cell chaining of the ^*ΔSS*^*nlpD ΔenvC* double mutant promoted by this variant is due to normal cell separation activity versus the lysis of cell units within the chains. Thus, the observed reduction of cell chaining may overestimate the separation activity of ^DsbA^NlpD^(27–379)^. Also, the cell lysis phenotype induced by this variant suggests that NlpD activation of AmiC is poorly regulated when NlpD is no longer bound to the OM. However, the observed degradation product of the periplasmic ^DsbA^NlpD^(27–379)^ variant may also contribute to the cell lysis phenotype, especially if it consists of the dLytM domain alone. Overall, we conclude that OM localization of NlpD is required for the efficient splitting of septal PG by its cognate amidase AmiC and possibly also for proper regulation of AmiC activity.

### Identification of factors required for NlpD activation using cell sorting

**Fig 5:**
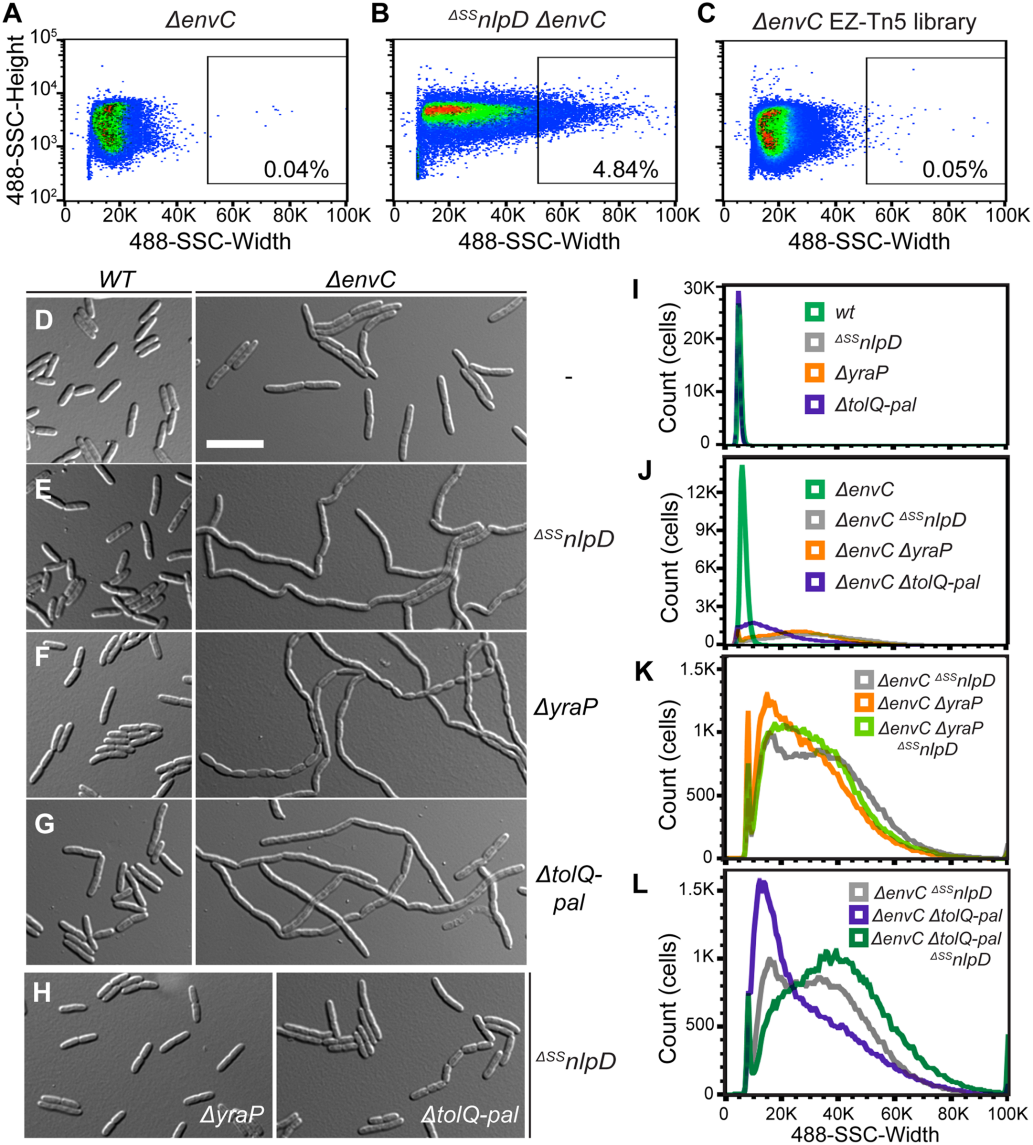
Flow cytometry-based enrichment and screen to identify potential NlpD regulators. **(A-C)** Dot plots of (**A**) a control non-chaining strain, TB140 (*ΔenvC*), (**B**) a control chaining strain, MT50 (^*ΔSS*^*nlpD ΔenvC*), and (**C**) the transposon (EZ-Tn5 <Kan-2>) mutagenized TB140 library, with side scatter pulse height (SSC-H; y-axis) versus side scatter pulse width (SSC-W; x-axis). The gate used for sorting is shown on the plot, with the number of events present in that gate indicated as a percentage of the total population. (**D-J**) Overnight cultures of (**D**) TB28 (*wt*), TB140 (*ΔenvC*), (**E**) MT47 (^*ΔSS*^*nlpD*), MT50 (*ΔenvC* ^*ΔSS*^*nlpD*), (**F**) MT140 (*ΔyraP*), MT135 (*ΔenvC ΔyraP*), (**G**) MT51 (*ΔtolQ-pal*), MT55 (*ΔenvC ΔtolQ-pal*), or (**H**) MT141 (^*ΔSS*^*nlpD ΔyraP*), MT53 (^*ΔSS*^*nlpD ΔtolQ-pal*) were diluted in LB medium. Cells were grown at 30°C to an OD_600_ of 0.3–0.5 before they were visualized on 2% agarose pads with DIC optics (**D-H**) or analyzed by flow cytometry (**I-J**). Bar = 10μm. (**K-L**) Overnight cultures of MT50 (*ΔenvC* ^*ΔSS*^*nlpD*) and (**K**) MT135 (*ΔenvC ΔyraP*) and MT179 (*ΔenvC ΔyraP* ^*ΔSS*^*nlpD*) or (**L**) MT55 (*ΔenvC ΔtolQ-pal*) and MT96 (*ΔenvC ΔtolQ-pal* ^*ΔSS*^*nlpD*) were diluted in LB medium. Cells were grown at 30°C to an OD_600_ of 0.15–0.25 before analysis by flow cytometry.

Just as FtsEX lies upstream of EnvC to connect its activation of the amidases to the activity of the divisome [13], regulators governing NlpD activity are also likely to exist. Moreover, given our observations that OM localization is required for proper NlpD function, we suspected that such regulators may be OM proteins. To identify these factors, we took advantage of the severe chaining phenotype caused by the simultaneous inactivation of both the EnvC and NlpD cell separation pathways. Based on SSC-W, long chains of the ^*ΔSS*^*nlpD ΔenvC* mutant can be effectively sorted from single *ΔenvC* mutant cells by gating for cells at the high end of the SSC-W distribution (Fig 5A and 5B). We therefore reasoned that mutants in any gene encoding a factor needed for NlpD activation should similarly display a severe chaining phenotype in an EnvC^-^ background, and that we could enrich for them by cell sorting.

For the enrichment and screen, an EnvC-depletion strain was transposon-mutagenized. The temperature-sensitive plasmid containing the inducible *envC* gene was cured during the mutagenesis procedure by growing the transposon mutants at 37°C [33]. The resulting transposon library in a *ΔenvC* background was then sorted for cells with high SSC-W (gate shown in Fig 5C). Sorted cells were plated on M9-maltose agar and grown at 37°C. A colony morphology screen was then used to distinguish mutants with severe chaining phenotype from non-chaining cells isolated as background. This secondary screen was based on the observation that colonies containing chaining cells are mucoid when grown on this medium at 37°C. Mucoid colonies were purified on LB and their cell chaining phenotype (as opposed to cell filamentation) confirmed microscopically (S5 Fig) before performing further analysis to map the position of the transposon insertion. As an indication that the screen was working properly, many of the transposon mutants had insertions mapping within *nlpD* or *amiC* (6 and 10 unique insertions, respectively). In addition to these expected mutants, many isolates had insertions that mapped to *tolB* (11 unique), *pal* (3 unique), *tolQ* (3 unique) and *tolA* (1 insertion). These genes encode members of the Tol-Pal system, which has been implicated in OM integrity and OM invagination during cell constriction [34–36]. Finally, three unique insertions were found within *yraP*, which encodes a predicted OM lipoprotein of unknown function [37].

To further investigate the potential connection of YraP and Tol-Pal with NlpD function, we assessed the division phenotypes of *ΔyraP* or *ΔtolQ-pal* mutants singly or in combination with *ΔenvC* or ^*ΔSS*^*nlpD* mutations. When grown in LB medium at 30°C, the single *ΔyraP* and *ΔtolQ-pal* mutants appeared to grow and divide normally (Fig 5F, 5G and 5I). However, when combined with an *envC* deletion, inactivation of YraP or Tol-Pal resulted in a severe chaining defect similar to the ^*ΔSS*^*nlpD ΔenvC* combination (Fig 5E–5G, 5J and S5C–S5E Fig). On the other hand, inactivating YraP or Tol-Pal in cells lacking NlpD had either no effect on division or caused only a mild cell separation defect (Fig 5H). The results are thus consistent with these factors being involved in the same pathway as NlpD, rather than EnvC, for cell separation. A triple ^*ΔSS*^*nlpD ΔenvC ΔyraP* mutant displayed a cell separation phenotype equivalent to the ^*ΔSS*^*nlpD ΔenvC* strain (Fig 5K). However, adding the *ΔtolQ-pal* lesion to a strain lacking NlpD and EnvC further exacerbated the already severe chaining defect of the double mutant (Fig 5L). The genetic results, therefore, suggest that YraP and Tol-Pal are required for proper NlpD function, with Tol-Pal likely playing a role that affects the cell separation activities of additional factors (see Discussion). Notably, consistent with their functional connection, YraP is well conserved among the Gammaproteobacteria and Betaproteobacteria, and its occurrence is correlated with the presence of an NlpD homolog (S6 Fig).

### NlpD and AmiC localize to the septal ring independently of YraP or the Tol-Pal system

**Fig 6:**
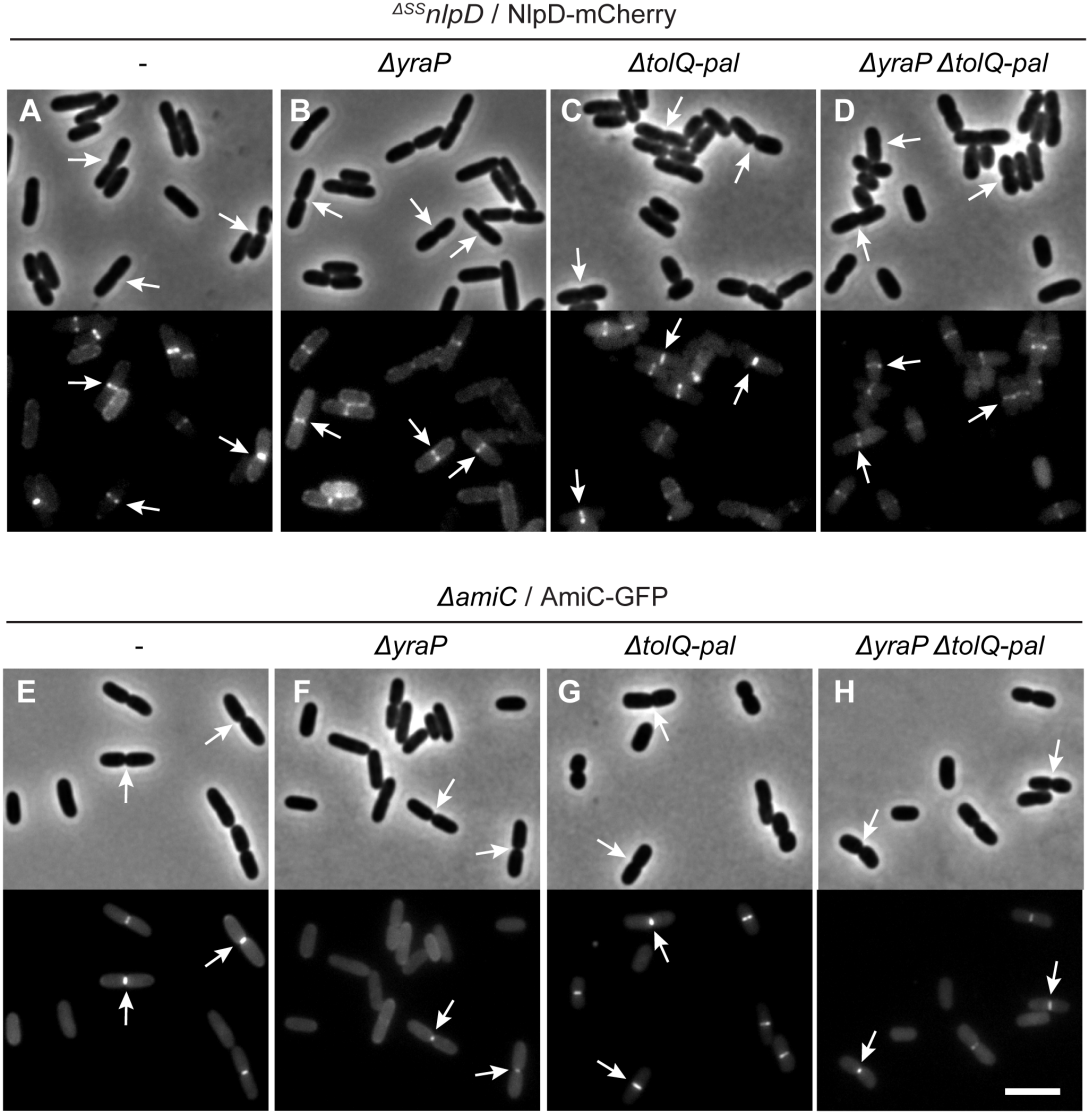
NlpD and AmiC localize to division sites independently of YraP and/or the Tol-Pal system. (**A-D**) Overnight cultures of (**A**) MT47 (^*ΔSS*^*nlpD*), (**B**) MT141 (^*ΔSS*^*nlpD ΔyraP*), (**C**) MT53 (^*ΔSS*^*nlpD ΔtolQ-pal*), or (**D**) MT147 (^*ΔSS*^*nlpD ΔtolQ-pal ΔyraP*) harboring the integrated expression construct attHKNP20 (*P*_*lac*_::*nlpD-mCherry*) were diluted in minimal M9-maltose medium supplemented with 100μM IPTG. (**E-H**) Overnight cultures of (**E**) TB143 (*ΔamiC*), (**F**) MT150 (*ΔamiC ΔyraP*), (**G**) MT149 (*ΔamiC ΔtolQ-pal*), or (**H**) MT178 (*ΔamiC ΔtolQ-pal ΔyraP*) harboring the integrated expression construct attHKNP16 (*P*_*lac-m3*_::*amiC-gfp*) were diluted in minimal M9-maltose medium supplemented with 25μM IPTG. For all strains, cells were grown at 30°C to an OD_600_ of 0.2–0.3 before they were visualized on 2% agarose pads by phase contrast and fluorescence microscopy. In all images, arrows indicate the localization of the protein fusion to division sites. Bar = 4μm.

A potential explanation for the YraP and Tol-Pal requirement for cell separation via the NlpD pathway is that one or both sets of factors is required for the recruitment of NlpD or AmiC to the division site. To investigate this possibility, we expressed either NlpD-mCherry or AmiC-GFP in cells lacking the corresponding native protein and inactivated for YraP, the Tol-Pal system, or both factors. Both fusions displayed a normal midcell localization pattern regardless of the genetic background (Fig 6), although the fluorescence signal of the NlpD-mCherry fusion appeared slightly reduced in the absence of both YraP and Tol-Pal (Fig 6D). Another mild effect observed was that the peripheral NlpD-mCherry signal was higher in *ΔyraP* cells relative to those with functional YraP (Fig 6B), and the midcell AmiC-GFP signal was slightly reduced (Fig 6F). We conclude that YraP and the Tol-Pal system are not required for the recruitment of NlpD and AmiC to the divisome, although YraP may enhance the septal localization of NlpD and AmiC.

### OM localization of YraP is important for its function

**Fig 7:**
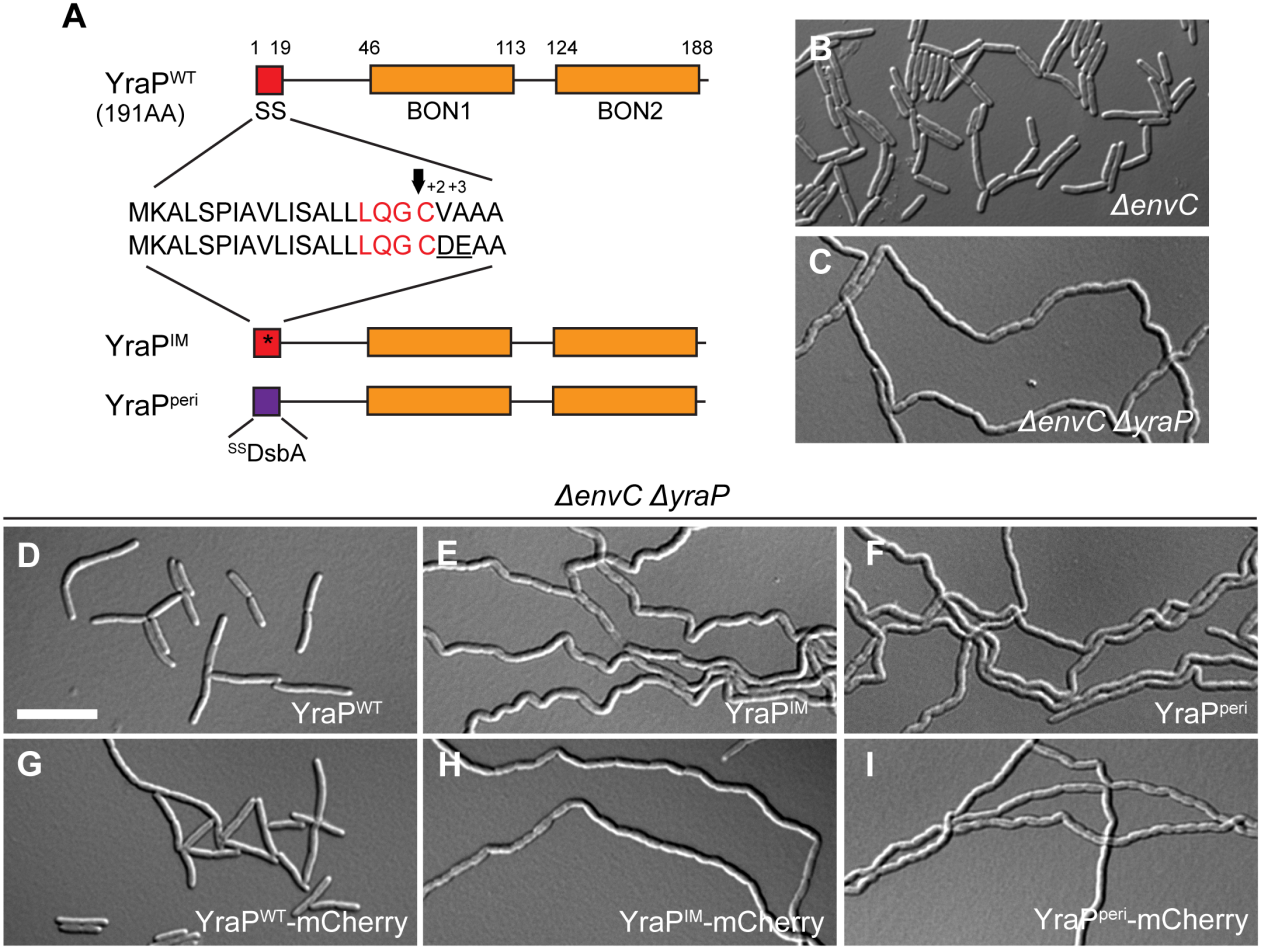
OM localization of YraP is required for cell separation in the absence of EnvC. (**A**) The domain structure of YraP is illustrated. Indicated are the signal sequence (SS; red) and the two **b**acterial **O**smY and **n**odulation domains (BON1/2; orange). Details of the signal sequence are presented with the lipobox in red and the arrow indicating the cleavage site just before the acylated cysteine. The IM-retained variant (YraP^IM^) contains a mutated signal sequence (indicated by the asterisk) with an aspartate and glutamate at the +2 and +3 positions after the acylated cysteine (underlined). The soluble periplasmic variant (YraP^peri^) is fused to the DsbA signal peptide (purple) that is cleaved upon export to the periplasm via the Sec system. (**B-I**) Overnight cultures of (**B**) TB140 (*ΔenvC*) or MT135 (*ΔenvC ΔyraP*) either (**C**) alone or harboring the integrated construct (**D**) attλMT196 (*P*_*lac*_::*yraP*^*WT*^), (**E**) attλMT198 (*P*_*lac*_::*yraP*^*IM*^), (**F**) attλMT209 (*P*_*lac*_::^*ss*^*dsbA-yraP*^*peri*^), (**G**) attλMT197 (*P*_*lac*_::*yraP*^*WT*^*-mCherry*), (**H**) attλMT199 (*P*_*lac*_::*yraP*^*IM*^*-mCherry*), or (**I**) attλMT210 (*P*_*lac*_::^*ss*^*dsbA-yraP*^*peri*^*-mCherry*) were diluted in minimal M9-maltose medium only (**B-C**) or supplemented with 10μM (**D, F-G, I**) or 20μM (**E, H**) IPTG. Cells were grown at 30°C to an OD_600_ of 0.2–0.25 before they were visualized on 2% agarose pads with DIC optics. Bar = 10μm.

YraP is predicted to be an outer membrane (OM) lipoprotein consisting of two bacterial OsmY and nodulation (BON) domains [38] (Fig 7A). To experimentally verify this localization, we used the cytological assay [32] employed above. Upon plasmolysis, wild-type YraP-mCherry displayed a smooth peripheral fluorescence signal consistent with an OM localization (S7A Fig). On the other hand, a variant of YraP-mCherry with the +2 and +3 positions changed to aspartate and glutamate to create a Lol-avoidance signal [31] followed the retracted IM in plasmolyzed cells (Fig 7A and S7B Fig). Swapping the normal signal sequence of YraP-mCherry for that of DsbA resulted in a fusion that showed a typical periplasmic localization, with fluorescence accumulating in the phase grey plasmolysis bays of shocked cells (Fig 7A and S7C Fig). These results strongly support the designation of YraP as an OM lipoprotein.

To assess the importance of subcellular localization for YraP function, we expressed the YraP variants described above, either untagged or with the C-terminal mCherry fusion, in the separation defective *ΔyraP ΔenvC* cells (Fig 7C). Both wild-type YraP variants rescued the chaining phenotype of *ΔyraP ΔenvC* such that cells only displayed the mild EnvC^-^ separation phenotype (Fig 7B, 7D and 7G). However, the YraP variants with modified subcellular localization (either IM or periplasmic) failed to complement the chaining phenotype of the *ΔyraP ΔenvC* strain (Fig 7E, 7F, 7H and 7I). We therefore conclude that YraP, like NlpD, needs to be OM-localized for its proper function.

### YraP is a new OM-localized component of the divisome

**Fig 8:**
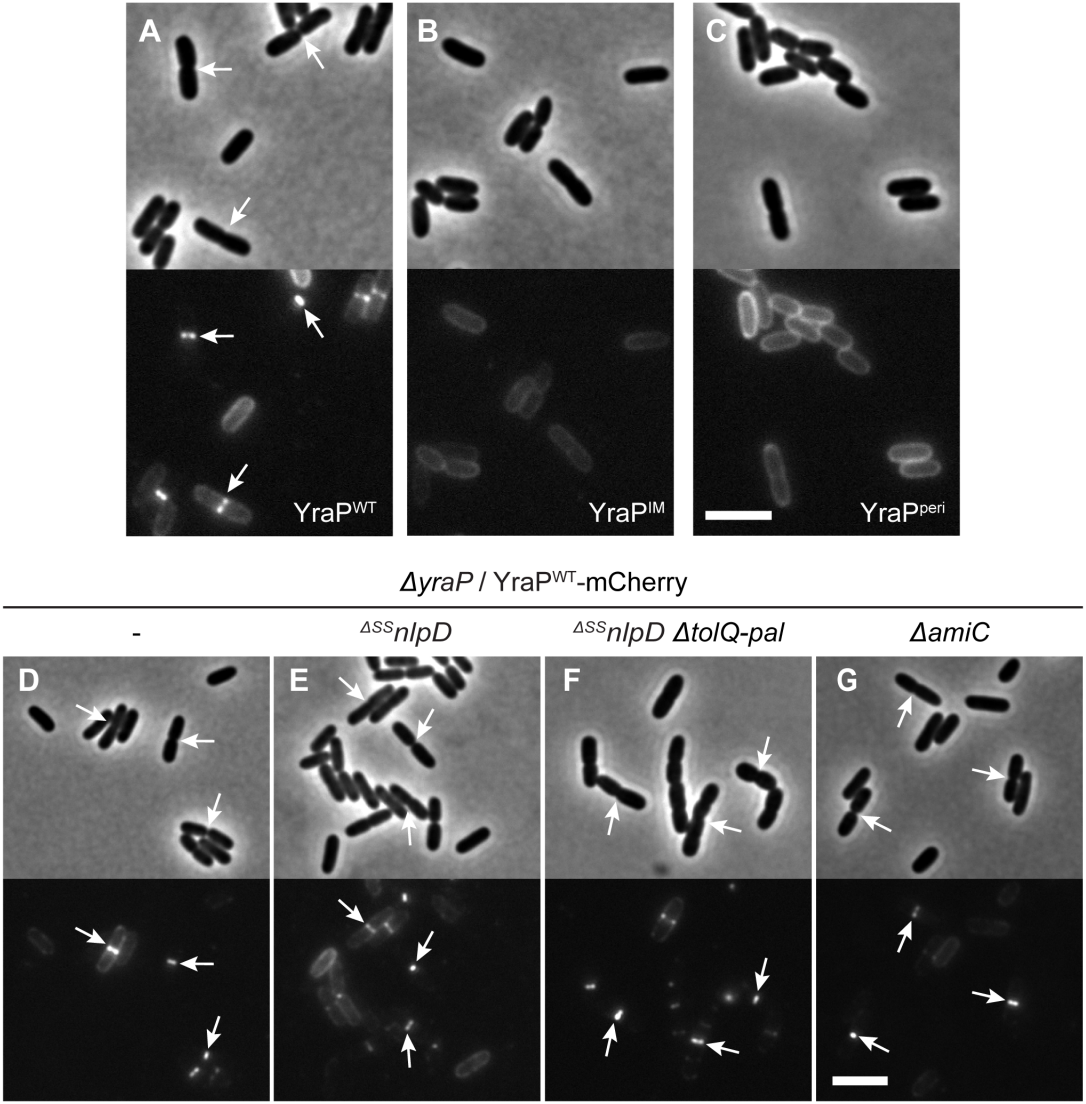
Recruitment of YraP to division sites is dependent on its OM localization but independent of NlpD, the Tol-Pal complex, and AmiC. (**A-C**) Overnight cultures of MT140 (*ΔyraP*) harboring the integrated construct (**A**) attλMT197 (*P*_*lac*_::*yraP*^*WT*^*-mCherry*), (**B**) attλMT199 (*P*_*lac*_::*yraP*^*IM*^*-mCherry*), or (**C**) attλMT210 (*P*_*lac*_::^*ss*^*dsbA-yraP*^*peri*^*-mCherry*) were diluted in minimal M9-maltose medium supplemented with 10μM (**A-C**) or 20μM (**B**) IPTG. (**D-G**) Overnight cultures of (**D**) MT140 (*ΔyraP*), (**E**) MT141 (*ΔyraP* ^*ΔSS*^*nlpD*), (**F**) MT147 (*ΔyraP* ^*ΔSS*^*nlpD ΔtolQ-pal*), and (**G**) AAY22 (*ΔyraP ΔamiC*) harboring the integrated construct attλMT197 (*P*_*lac*_::*yraP*^*WT*^*-mCherry*) were diluted in minimal M9-maltose medium supplemented with 10μM IPTG. All cultures were grown at 30°C to an OD_600_ of 0.15–0.2 before cells were visualized on 2% agarose pads by phase contrast and fluorescence microscopy. Arrows indicate localization of the protein fusion to division sites. Bar = 4μm.

**Fig 9:**
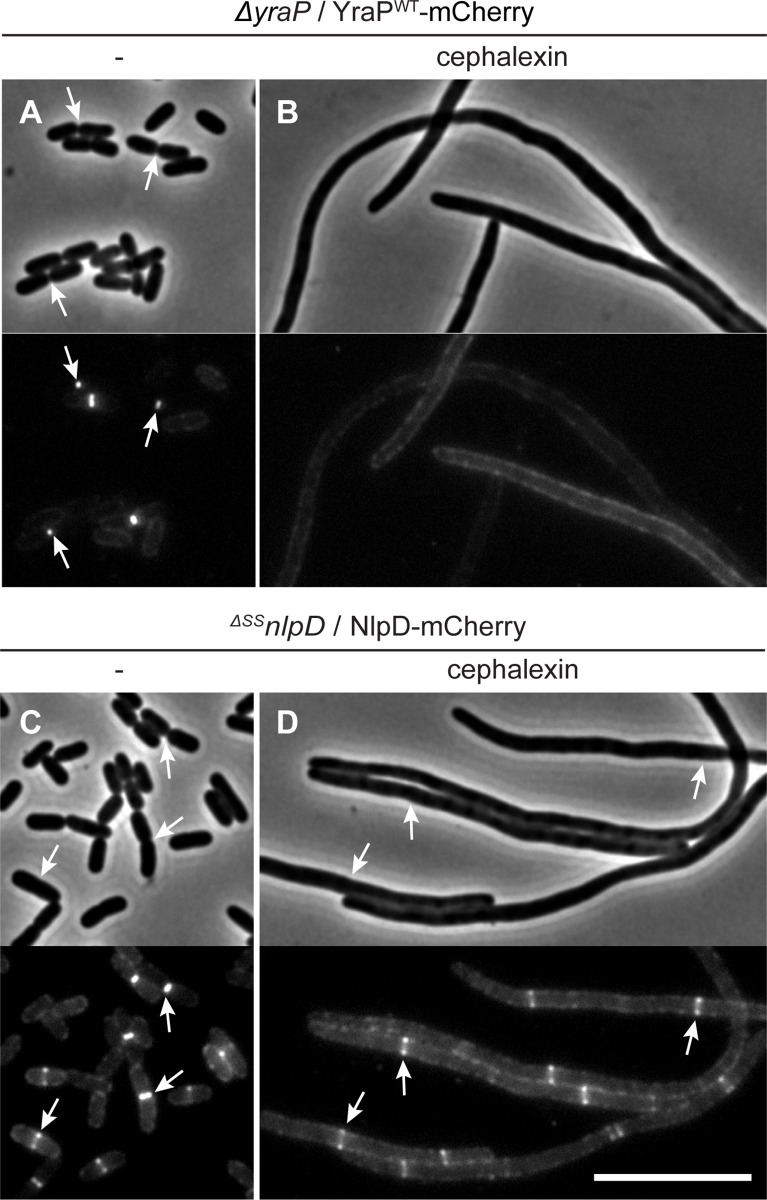
Localization of YraP or NlpD in cephalexin-treated cells. Overnight cultures of (**A-B**) MT140 (*ΔyraP*) harboring the integrated construct attλMT197 (*P*_*lac*_::*yraP-mCherry*) or (**C-D**) MT47 (^*ΔSS*^*nlpD*) harboring the integrated expression construct attHKNP20 (*P*_*lac*_::*nlpD-mCherry*) were diluted in minimal M9-maltose medium supplemented with either 25μM (**A-B**) or 100μM (**C-D**) IPTG and grown at 30°C until mid-log. Cultures were then backdiluted into M9-maltose medium with the indicated IPTG concentration with or without 10μg/ml cephalexin as indicated. Cells were grown at 30°C to an OD_600_ of 0.2 before they were visualized on 2% agarose pads by phase contrast and fluorescence microscopy. Arrows indicate localization of the protein fusion to division sites. Bar = 10μm.

The genetic results implicate the Tol-Pal system and YraP in the pathway that stimulates AmiC activation by NlpD. All of these factors except YraP have been shown to be recruited to the divisome. We therefore investigated the subcellular localization of YraP. In support of the protein playing a direct role in the division process, a functional YraP-mCherry fusion protein displayed a band-like localization pattern at midcell in cells with a visible constriction (Fig 8A). The IM-retained and periplasmic variants of YraP-mCherry both displayed a dispersed peripheral localization signal (Fig 8B and 8C). These fusions were found to be intact and expressed to comparable protein levels to the wild-type fusion protein by immunoblotting (S7D Fig), indicating that OM localization is required for YraP recruitment to the divisome. Recruitment of YraP-mCherry to the division site was independent of NlpD, the Tol-Pal system, and AmiC (Fig 8E–8G). As expected, however, YraP-mCherry did not localize to potential division sites in cells producing the FtsZ antagonist SulA that blocks divisome formation (S8A and S8B Fig). Notably, unlike NlpD-mCherry, which localizes to divisome assemblies in cells blocked for division by the PBP3 (FtsI) inhibitor cephalexin, YraP-mCherry failed to localize in similarly treated cells (Fig 9). The recruitment pathway for all essential divisome components remains functional in cephalexin treated cells [39], but constriction is blocked due to the disruption of septal cell wall biogenesis caused by the drug. Thus, YraP likely requires the initiation of constriction for its localization. A similar requirement was previously demonstrated for AmiC [40,41].

### Inactivation of NlpD results in OM defects

**Fig 10:**
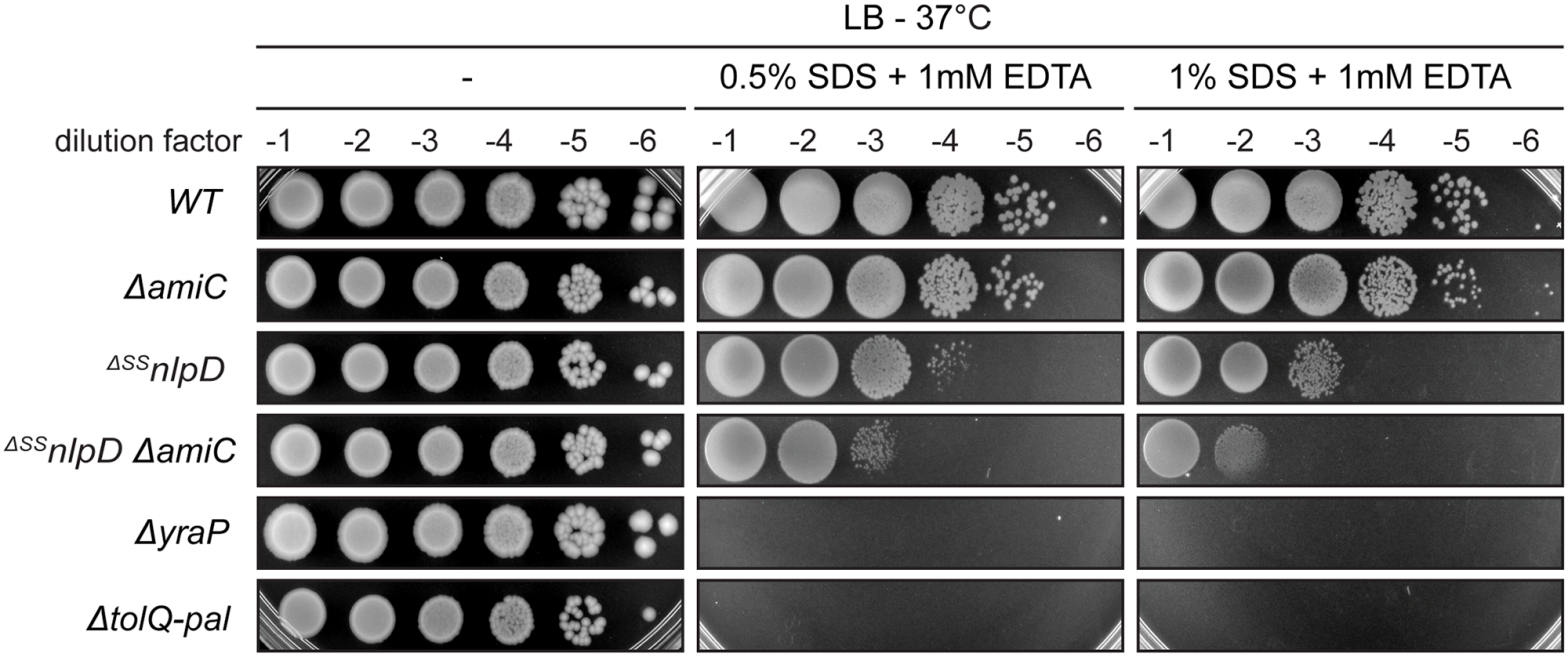
NlpD inactivation results in OM integrity defects. Cells of TB28 (*WT*), TB143 (*ΔamiC*), MT47 (^*ΔSS*^*nlpD*), MT176 (^*ΔSS*^*nlpD ΔamiC*), MT140 (*ΔyraP*), and MT51 (*ΔtolQ-pal*) were grown overnight in LB at 30°C. Following normalization for cell density (OD_600_ = 1), the resulting cultures were serially diluted (10^−1^ to 10^−6^), and 5 μl of each dilution was spotted on LB agar only or supplemented with the indicated concentrations of SDS and EDTA. The plates were incubated at 37°C and photographed after ~24 hours.

The observed midcell recruitment of YraP places it within the divisome, along with the Tol-Pal system [36], NlpD [7], and AmiC [41], and suggests that all of these factors are directly participating in the envelope remodeling process at the division site. Although the precise function of the Tol-Pal system and YraP remains unclear, the defects in OM integrity observed upon their inactivation [37,42,43] indicate that they play critical roles in OM physiology during the formation of new daughter cell poles. Our genetic analysis suggests that these divisome components are required for the cell separation activity of NlpD. We therefore wondered whether a defect in NlpD and/or its cognate amidase AmiC might contribute to the OM problems observed when the Tol-Pal system or YraP are crippled. Notably, although the defect was not as severe as that observed with cells lacking YraP or the Tol-Pal system, ^*ΔSS*^*nlpD* cells were found to be hypersensitive to treatment with a combination of the detergent sodium-dodecyl sulphate (SDS) and the metal-chelating agent ethylenediaminetetraacetic acid (EDTA), a phenotype indicative of OM defects [44] (Fig 10). Moreover, a *ΔamiC* mutant does not show a similar hypersensitivity phenotype, indicating that the role of NlpD in maintaining the OM permeability barrier is likely distinct from its function in activating amidase activity for septal PG splitting. However, inactivation of AmiC in ^*ΔSS*^*nlpD* cells further exacerbated their SDS-EDTA sensitivity, suggesting some contribution of AmiC to OM integrity in the absence of NlpD. This observation suggests that AmiC may be cross activated by other dLytM factors and that its deletion in ^*ΔSS*^*nlpD* cells further reduces cell separation efficiency, defects in which are well known to result in reduced OM barrier function [10–12,45]. Taken together, the multiple connections between NlpD activity and the OM uncovered in this study support a model in which NlpD provides a key link that helps coordinate OM invagination and PG remodeling at the division site.

## Discussion

**Fig 11:**
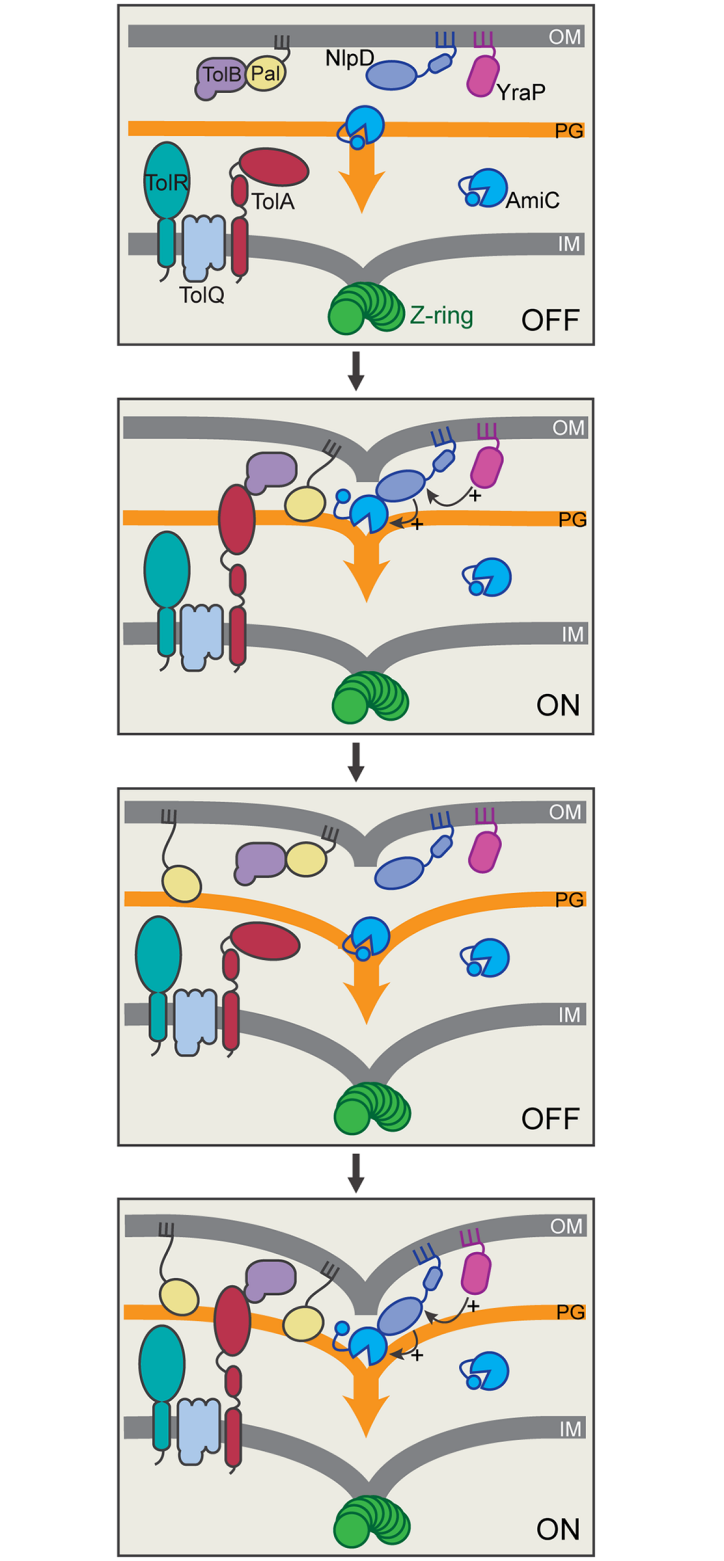
Model for the regulation of NlpD during cell division. Shown is a schematic depicting two cycles of a potential coupling mechanism coordinating septal PG splitting by NlpD/AmiC with OM invagination. Prior to the initiation of constriction, we envision that the dLytM domain of OM-anchored NlpD is prevented from accessing the PG layer and/or AmiC, potentially by physical distance or protein conformation constraints. When the divisome is activated, OM constriction promoted by the Tol-Pal system may bring the dLytM domain of NlpD into proximity of the PG layer and AmiC where it can stimulate septal PG splitting. AmiC activation also appears to require YraP, which may activate NlpD directly or indirectly through its yet to be determined role in maintaining OM integrity. PG processing by AmiC is expected to increase the distance between the OM and PG layer, thus returning NlpD to its inactive configuration and requiring another round of OM constriction to trigger further PG processing and so on until cell division is complete and the daughter cells are separated. Shown are hypothetical protein conformations and interactions within the Tol-Pal system based on current literature [65–71].

Cell division in gram-negative bacteria involves the coordinated constriction of all three cell envelope layers. Surprisingly little is known about the factors and mechanisms that mediate this coordination, given its importance for maintaining the OM permeability barrier that promotes drug resistance of these cells. Here, we have investigated the activation of cell wall remodeling at the division site by the OM lipoprotein NlpD. In doing so, we have uncovered several critical links between its activity and the OM that suggest a key role for NlpD in coupling the processes of OM invagination and septal PG splitting during cytokinesis (Fig 11).

The initial connection between NlpD activity and the OM came with the observation that NlpD must be transported to the OM for it to function properly. If retained in the IM, it is unable to activate septal PG splitting despite being recruited to midcell. Also, release of NlpD from the membrane and its production as a soluble periplasmic protein renders it less functional at cell separation and results in an elevated frequency of cell lysis. This lytic phenotype is dependent on AmiC, suggesting that NlpD is capable of activating the amidase in the periplasm, but does so in a poorly controlled manner, leading to inefficient splitting of the septal PG and cell wall damage. When purified, a soluble version of NlpD lacking its lipid-anchor is also capable of significantly stimulating PG degradation by AmiC *in vitro* [6]. The reaction appears to be unregulated and no additional activation factors are required to observe the AmiC stimulation. Thus, the combined *in vitro* and *in vivo* results imply that localization of NlpD to the OM restrains its activity, most likely by promoting the adoption of an inactive conformation of the protein, either alone or in complex with additional factors.

The nature of the regulated conformation of NlpD remains unclear, but one attractive possibility is that OM-localized NlpD is held at a distance from the PG layer such that its dLytM domain is unable to physically “reach” the matrix to stimulate AmiC where it is in position to cleave PG. Because NlpD possesses two relatively long linker domains predicted to be unstructured, OM-localization alone is unlikely to be sufficient to keep NlpD from accessing the PG layer. However, it is becoming increasingly apparent that OM lipoproteins are not always simply tethered to the OM by their lipid anchor. Lipoproteins like Lpp and RcsF can adopt transmembrane topologies and/or be assembled in complexes with OM porins [46–49]. Indeed, NlpD from *Haemophilus influenzae* has been detected at the cell surface [50], most likely due to it adopting a complex OM topology that exposes portions of the protein to the surface. Thus, although the exact configuration of NlpD in the OM requires investigation, its localization to the membrane appears to be critical for its proper regulation, potentially by holding the dLytM domain at a distance or otherwise sequestering it from the PG layer and/or AmiC (Fig 11).

Regardless of the precise regulated state of NlpD in the OM, it is reasonable to assume that it is responsive to the functional status of the divisome such that it only becomes activated to stimulate PG cleavage by AmiC at the appropriate time during the division cycle. Our genetic results indicate that the Tol-Pal system and the YraP protein are likely to be among the divisome components that mediate this activation. In this regard, the physical restriction model for NlpD control in which its dLytM domain is held at a distance from the PG layer suggests a simple mechanism by which the Tol-Pal system might stimulate cell separation by NlpD (Fig 11). Cells inactivated for the Tol-Pal system release OM vesicles that appear to be preferentially shed from the division site [36]. Based on this phenotype and the tight localization of the system to the divisome, it has been proposed that the transenvelope Tol-Pal complex promotes the constriction of the OM during division [36]. According to the model, OM retraction by the Tol-Pal system at the division site brings the Pal lipoprotein into contact with the PG layer to temporarily anchor the membrane to the cell wall until more permanent Lpp linkages can be established [36]. A similar mechanism may be at work with regard to NlpD activation in which the OM constriction activity of the Tol-Pal system moves the dLytM domain of NlpD into proximity of the PG layer where it can stimulate septal PG hydrolysis by AmiC (Fig 11). An attractive feature of this model for NlpD regulation is that it does not require a direct physical interaction between NlpD and the Tol-Pal complex as we have been unable to reliably detect one despite numerous attempts. Furthermore, such a regulatory mechanism would allow the Tol-Pal system to simultaneously control the activities of a diverse set of OM lipoproteins, many of which have PG remodeling capabilities that might be limited by their physical distance from the PG layer. Such a broad regulatory role in controlling the PG remodeling activities of lipoproteins may partly explain the pleiotropic phenotype of *tol-pal* mutants and the observation that the system likely affects the cell separation activities of factors beyond just NlpD (Fig 5H and 5L). Interestingly, the Tol-Pal system has also been implicated in the modulation of PG synthesis at the division site [51], suggesting that, like FtsEX [18], it might serve to coordinate PG synthesis and hydrolysis activities at the septum.

Relative to the Tol-Pal system, much less is known about the function of the YraP protein. Consequently, it is more difficult to predict how it might be involved in promoting the cell separation activity of NlpD. Previous results have implicated YraP in OM integrity and its inactivation has been shown to result in defects in the OM assembly of some proteins [37,52]. Here, we have connected YraP with NlpD function, definitively demonstrated its OM localization, and showed that it must be OM-localized to promote cell separation. Furthermore, we discovered that YraP is recruited to the divisome, suggesting it is likely to be directly participating in the division process. Notably, unlike NlpD, YraP fails to be recruited to division sites in cephalexin-treated cells. This result indicates that YraP most likely requires cell constriction for its localization to the divisome. Thus, YraP presumably arrives at the divisome after NlpD and could conceivably be part of a mechanism by which divisome activation is communicated to NlpD to promote its activation. However, we were unable to detect an interaction between YraP and NlpD in a purified system, suggesting that the activation mechanism may be indirect and related to the role of YraP in OM function.

Further work will be required to determine the precise roles of YraP and the Tol-Pal system in NlpD activation. Nevertheless, their requirement for proper function of the NlpD cell separation pathway indicates that NlpD not only requires OM localization for proper activity, but that its activation is also responsive to the status of the OM. The converse is also true. Growth assays on SDS-EDTA medium indicate that NlpD is required for proper OM integrity via a mechanism that is AmiC-independent and apparently distinct from its function in septal PG splitting. Thus, taken together, the results presented in this report make a strong case for NlpD functioning as a key mediator between OM constriction and PG remodeling that helps coordinate these processes during cytokinesis. Such a role for LytM-containing proteins may be conserved as another LytM factor, DipM, has been implicated in the coordination of PG remodeling and OM constriction in *Caulobacter crescentus* [26–28]. However, the molecular details of the coupling mechanism may differ in *E*. *coli* and *C*. *crescentus* since NlpD is an OM-linked lipoprotein whereas DipM is soluble in the periplasm. Furthermore, neither NlpD, Tol-Pal, nor YraP is essential for growth or division of *E*. *coli*, indicating that additional factors with overlapping activities are also likely to be involved. Continued study of the envelope remodeling events orchestrated by the divisome in a variety of bacterial species are therefore likely to reveal new weaknesses in the process and provide novels targets for the disruption of the OM permeability barrier and sensitization of gram-negative cells to antibiotics.

## Materials and methods

### Media, bacterial strains, and plasmids

Cells were grown in LB (1% tryptone, 0.5% yeast extract, 0.5% NaCl) or minimal M9 medium [53] supplemented with 0.2% casamino acids and 0.2% maltose. Unless otherwise indicated, antibiotics were used at concentrations of 25 μg/ml (chloramphenicol; Cm), 25 μg/ml (kanamycin; Kan), 5 μg/ml (tetracycline; Tet), or 50 μg/ml (ampicillin, Amp).

The bacterial strains used in this study are listed in S1 Table. All *E*. *coli* strains used in the reported experiments are derivatives of MG1655 [54]. All deletion alleles were either sourced from the Keio knockout collection [55] or constructed to resemble those in the collection, with the exception of the *ΔtolQ-pal* and ^*ΔSS*^*nlpD* alleles. Plasmids used in this study are listed in S2 Table. Plasmid construction details for this study are provided in S1 Text. Plasmids that are derivatives of CRIM vectors [56] were integrated into phage attachment sites (HK022 or λ) using the helper plasmids pTB102 [57] or pINT-ts [56], respectively, as described [56]. PCR was performed using either KOD polymerase (Novagen) or Q5 polymerase (NEB) according to the manufacturer’s instructions. Unless otherwise indicated, MG1655 chromosomal DNA was used as the template. Plasmid DNA and PCR fragments were purified using the Zyppy plasmid miniprep kit (Zymo Research) or the Qiaquick PCR purification kit (Qiagen), respectively. Sequencing reactions were carried out with an ABI3730xl DNA analyzer at the DNA Resource Core of Dana-Farber/Harvard Cancer Center (funded in part by NCI Cancer Center support grant 2P30CA006516-48).

### Recombineering

Since the promoter of *rpoS* is present within the coding sequence of *nlpD*, a gene knockout of *nlpD* is polar on *rpoS* expression. Therefore, in order to make an *nlpD* null strain, the N-terminal lipoprotein signal sequence of NlpD was deleted (^*ΔSS*^*nlpD*) to prevent the export of this protein to the periplasm, where it normally functions. The ^*ΔSS*^*nlpD*::*kan*^*R*^ allele was constructed by replacing the region between the 2nd codon and 27th codon of *nlpD* with the Kan^R^ cassette by λ recombineering as described previously [55,58]. The Kan^R^ cassette was amplified from pKD13 [59] using the primers 5’-TGTCACTGGTTATTAACCAATTTTTCCTGGGGGATAAATGATTCCGGGGATCCGTCGACC-3’ and 5’-TAACGGAGCTGACCGGTGCCGGTGGATTTGAAGTGTCAGATGTAGGCTGGAGCTGCTTCG-3’. The resulting PCR product was electroporated into strain TB10, and recombinants were selected at 30°C on LB with kanamycin, generating a chromosomal deletion as described previously [60]. The Kan^R^ cassette was removed using FLP recombinase expressed from pCP20 [59], leaving behind the *frt* site scar to replace the signal sequence of ^*ΔSS*^*nlpD*.

The *ΔtolQ-pal*::*Kan*^*R*^ allele was constructed by replacing the region between the 2nd codon of *tolQ* and the 7th codon from the stop codon of *pal* with the *Kan*^*R*^ cassette by λ recombineering as described previously [55,58]. The *Kan*^*R*^ cassette was amplified from pKD13 [59] using the primers 5’-GTGCGCTTCCCAAGTCTATTGTCGCGGAGTTTAAGCAGTGATTCCGGGGATCCGTCGACC-3’ and 5’-TACTGCTCATGCAATTCTCTTAGTAAACCAGTACCGCACGTGTAGGCTGGAGCTGCTTCG-3’. The resulting PCR product was purified and electroporated into strain TB28/pKD46 as described previously [29], and recombinants were selected at 30°C on LB plates supplemented with kanamycin to generate the chromosomal deletion.

The *ΔyraP*::*Kan*^*R*^ allele was constructed by replacing the region between the 2nd codon and the 7th codon from the stop codon of *yraP* with the *Kan*^*R*^ cassette as described above. The *Kan*^*R*^ cassette was amplified from pKD13 using the primers 5’-GTGCGCTTCCCAAGTCTATTGTCGCGGAGTTTAAGCAGTGATTCCGGGGATCCGTCGACC-3’ and 5’-TACTGCTCATGCAATTCTCTTAGTAAACCAGTACCGCACGTGTAGGCTGGAGCTGCTTCG-3’. The resulting fragment was electroporated into strain TB10 and the recombinants were selected at 30°C on LB plates with kanamycin, generating a chromosomal deletion as described previously [60].

The *ΔtolQ-pal*::*Kan*^*R*^ and *ΔyraP*::*Kan*^*R*^ alleles were transferred between strains by P1 transduction. The P1 lysate for *ΔtolQ-pal*::*Kan*^*R*^ was made on a strain with *ΔtolQ-pal*::*Kan*^*R*^ and carrying pMT94 (P_*lac*_::*nativeRBS_ybgC_tolQRA*) to express the *tolQRA* genes. When necessary, the Kan^R^ cassette was removed using FLP recombinase expressed from pCP20 [59].

### Plasmolysis

Overnight cultures were backdiluted in M9 medium with 0.2% maltose and IPTG and grown at 30°C (see figure legends for specific IPTG concentrations and final culture OD_600_). Cells were harvested by centrifugation, washed in M9-maltose once, resuspended in plasmolysis buffer (15% sucrose, 25mM HEPES, pH 7.5, 20mM sodium azide), and immediately imaged on 1% agarose pads containing 15% sucrose using phase contrast and fluorescence microscopy as described below.

### Membrane staining with FM 4-64FX

Cells were grown in LB medium at 30°C to an OD_600_ of 0.2–0.3. To 1ml of culture, the membrane dye FM 4-64FX was added to a final concentration of 5 μg/ml, cells were then incubated for 10 min in the dark, followed by fixation with 2.5% formaldehyde and 0.04% gluteraldehyde in 60mM sodium phosphate buffer, pH 7.4, for 20 min at room temperature. After 3 washes with PBS (137mM NaCl, 2.7mM KCl, 1mM CaCl_2_, 0.5mM MgCl_2_, 10mM Na_2_PO_4_, 1.8mM KH_2_PO_4_, pH 7.4), the cells were immediately imaged on pads made with 2% agarose in LB using phase contrast and fluorescence microscopy as described below.

### Microscopy

Cells were imaged on 2% agarose pads containing 1X M9 salts (unless indicated otherwise) using a Nikon TE2000 inverted microscope outfitted with a Nikon Intensilight illuminator, a Coolsnap HQ2 CCD camera from Photometrics, a Nikon CFI Plan Apo VC 100x objective (1.4 NA) for differential interference contrast (DIC) imaging or a CFI Plan Apo DM 100x objective (1.4 NA) for phase contrast imaging. Please see figure legends for growth conditions used for specific experiments. Filter cubes for fluorescence image acquisition were from Chroma. mCherry and GFP fluorescence images were taken using the ET-mCherry filter set (Chroma 49008) and ET-GFP filter set (Chroma 49002), respectively. Images were captured using Nikon Elements software, exported and cropped for figure preparation in MetaMorph (Molecular Devices).

### Protein purification

NlpD was overexpressed and purified with a 6xHis-SUMO (H-SUMO) tag fused to its N-terminus [61,62]. The sequence of the affinity tag was MRGSHHHHHHMASG. The SUMO sequence was amplified from the *Saccharomyces cerevisiae genome* (gene Smt3) [63]. H-SUMO-NlpD(27–379) was purified from BL21(λDE3)/pTU119. Overnight cultures were diluted 1:200 into 1 L of LB supplemented with ampicillin (50 μg/ml) and cells were grown at 30°C to an OD_600_ of 0.5. IPTG was then added to 1 mM, growth was continued for an additional 3.5 hours, and the cells were harvested by centrifugation. Cell pellets were resuspended in 25 ml Buffer A (20 mM Tris-HCl, pH 7.4, 500 mM NaCl, 20 mM imidazole) and stored at -80°C prior to use for protein purification. For purification, the frozen resuspension was thawed, and the cells were disrupted by passing them through a French pressure cell twice at 15,000 psi. Cell debris were pelleted by centrifugation at 17,000 x g for 10 minutes at 4°C. The clarified lysate was incubated with Ni-NTA agarose beads (Qiagen) for 1 hour at 4°C and then loaded on a gravity flow column (BioRad). The column was washed with 10ml Buffer A and the H-SUMO-NlpD(27–379) was eluted with Buffer B (20 mM Tris-HCl, pH 7.4, 500 mM NaCl, 300 mM imidazole). Peak fractions were collected and dialyzed against 1 L of buffer D (50 mM Tris-HCl, pH 7.4, 300 mM NaCl, 10% glycerol). After purification of the H-SUMO fusion protein, the H-SUMO tag was removed using 6xHis-tagged SUMO protease (H-SP) [63]. Cleavage reactions were passed through Ni-NTA resin (Qiagen) to remove free H-SUMO and H-SP, yielding a pure preparation of untagged NlpD without added non-native residues. Aliquots of both tagged and untagged versions of NlpD(27–379) were stored at -80°C prior to use.

### Antibody preparation and affinity purification

Rabbit polyclonal antibodies against purified NlpD were prepared by Covance. Tagged NlpD protein prepared as described above was used in a custom antibody production service. The anti-NlpD antibodies were subsequently affinity-purified using purified untagged NlpD conjugated to AminoLink Plus resin (Pierce), according to the manufacturer’s instructions.

### Immunoblotting

Strains were grown as described in the figure legends. At the designated times, cells were harvested and whole-cell extracts were prepared as described previously [64]. The protein concentration of each extract was determined using the Non-Interfering protein assay (G-Biosciences) according to the manufacturer’s instructions. Protein concentrations were normalized between extracts and the indicated amount of total protein from each extract was separated on a 4–20% Mini-PROTEAN^®^ TGX precast protein gel (BioRad) or 12% polyacrylamide gel. Proteins were transferred to a PVDF membrane (Whatman) and the membrane was blocked with 2–5% skim milk in TBS-T (10mM Tris HCl, pH 7.5, 100mM NaCl, 0.1% Tween 20). The primary antibodies were used at the dilutions of either 1:5,000 for anti-mCherry or 1:10,000 for the affinity-purified anti-NlpD antibody. To reduce non-specific binding, the anti-mCherry primary antibody solution was pre-incubated with 100μL of TB28 (wt) cell lysate (OD_600_ of ~15) prior to application to the membrane. The secondary goat anti-rabbit antibody conjugated to horseradish peroxidase (Rockland) was used at the dilution of 1:40,000, and the blot was developed using the Super Signal West Pico system (Pierce) according to the manufacturer’s protocol. Chemiluminescence was detected using a BioRad Chemidoc system.

### Transposon mutagenesis

Mutagenesis of a *ΔenvC* strain was inefficient, most likely due to the cell division defects displayed by these cells. Instead, an EnvC depletion strain TB140/pMT187 [*ΔenvC/Plac*::*envC-LE*] was mutagenized with the Ez-Tn5 <Kan-2> transposome (Epicentre) as previous described [29]. Mutants were selected at 37°C on minimal M9 containing 50 μg/ml kanamycin, yielding a library of ~ 50,000 independent transposon insertions. Growth at 37°C resulted in the loss of the pMT187 plasmid (through combined effects of its temperature-sensitive origin of replication and cleavage by I-Sce-I [33]. The resulting *ΔenvC* mutant library was pooled as a slurry, and aliquots were stored frozen at -80°C.

### Flow cytometry analysis

The cell chaining phenotype of different strains was assessed using flow cytometry. Cells were harvested by a low speed centrifugation (2,500 x g for 2 min), resuspended in PBS to an OD_600_ ~ 0.1–0.2 and filtered through a 70 μm filter before analysis. Flow cytometry analysis was carried out on a MoFlo Astrios EQ (Beckman Coulter) using a 70μm nozzle, at the Division of Immunology’s Flow Cytometry Facility at Harvard Medical School. Flow cytometry histograms of cells (~ 100,000 cells for each sample) with increasing side scatter pulse width (SSC-W) were plotted using FlowJo.

### Flow cytometry-based enrichment

For the control strains, overnight cultures were diluted in LB medium and grown at 30°C to an OD_600_ of 0.2–0.3. For the transposon mutagenized *ΔenvC* library, a frozen aliquot was thawed, back-diluted twice in LB and grown at 30°C to an OD_600_ of 0.3. The cells were then harvested by a low spin centrifugation (1,000 x g for 1–2 min), resuspended in PBS to an OD_600_ ~ 0.1–0.2 and filtered through a 70μm filter before analysis and sorting. Cell resuspensions were analyzed and sorted with a 100μm nozzle on the MoFlo Astrios EQ (Beckman Coulter) described above. Gates for sorting were defined on a side scatter pulse height (SSC-H; y-axis) versus side scatter pulse width (SSC-W; x-axis) dot plot. Sorted cells were plated on minimal M9-maltose plates and incubated at 37°C overnight. Mucoid colonies were purified on LB, the chaining phenotype confirmed by phase contrast microscopy, and the sites of the transposon insertions were identified by arbitrarily primed PCR followed by sequencing [29].

### Bioinformatic analysis of YraP and NlpD distribution

Primary protein sequences of *E*. *coli* YraP and NlpD were used as queries in a BLASTP search against the 201602 update of NCBI nr database. The E-value cutoff for hits was set at 1.00 x 10^−20^. The phylogenetic tree was produced via PhyloT webtool (http://phylot.biobyte.de), using an abridged version of the NCBI Representative Genomes database. The tree and the plots of YraP and NlpD distributions were then rendered with iToL webtool (http:://itol.embl.de).

## Supporting information

S1 Fig NlpD truncations are expressed and mostly intact. (**A**) Cells of MT47 (^*ΔSS*^*nlpD*) alone or expressing different NlpD-mCherry fusions from the integrated constructs attHKNP20 (*P*_*lac*_::*nlpD*^*WT*^*-mCherry*), attHKMT101 (*P*_*lac*_::*nlpD*^*(1–189)*^*-mCherry*), attHKMT103 (*P*_*lac*_::*nlpD*^*(1–115)*^*-mCherry*), attHKMT178 (*P*_*lac*_::^*ss*^*dsbA-nlpD*^*(102–175)*^*-mCherry*), attHKMT180 (*Pl*_*ac*_::^*ss*^*dsbA-nlpD*^*(250–379)*^*-mCherry*), attHKMT182 (*P*_*lac*_::^*ss*^*dsbA-nlpD*^*(189–379)*^*-mCherry*), or attHKMT149 (*P*_*lac*_::*nlpD*^*(1–30)*^*-mCherry*) were diluted in minimal M9-maltose medium supplemented with IPTG at concentrations indicated in Fig 2, grown at 30°C, and then harvested for whole-cell extract preparation. (**B**) Cells of MT50 (^*ΔSS*^*nlpD ΔenvC*) alone or expressing different NlpD variants from the integrated constructs attHKMT20 (*P*_*lac*_::*nlpD*^*WT*^), attHKMT102 (*P*_*lac*_::*nlpD*^*(1–189)*^), attHKMT104 (*P*_*lac*_::*nlpD*^*(1–115)*^), attHKMT179 (*Pl*_*ac*_::^*ss*^*dsbA-nlpD*^*(250–379)*^), or attHKMT181 (*P*_*lac*_::^*ss*^*dsbA-nlpD*^*(189–379)*^) were diluted in minimal M9-maltose medium alone (MT50) or supplemented with either 150μM (attHKMT20, attHKMT179, or attHKMT181) or 1mM (attHKMT102 or attHKMT104) IPTG, grown at 37°C, and then harvested for whole-cell extract preparation. Proteins in the resulting extracts were separated by SDS-PAGE, transferred to PVDF, and NlpD was detected with anti-mCherry (**A**) or affinity-purified anti-NlpD (**B**) antibodies. The arrows indicate the NlpD variant present in each strain. The asterisks denote possible degradation products of each variant.
(TIF)

S2 Fig Only full-length NlpD properly promotes cell separation. (**A-E**) Overnight cultures of MT50 harboring the integrated expression constructs (**A**) attHKMT20 (*P*_*lac*_::*nlpD*^*WT*^), (**B**) attHKMT102 (*P*_*lac*_::*nlpD*^*(1–189)*^), (**C**) attHKMT104 (*P*_*lac*_::*nlpD*^*(1–115)*^), (**D**) attHKMT179 (*Pl*_*ac*_::^*ss*^*dsbA-nlpD*^*(250–379)*^), or (**E**) attHKMT181 (*P*_*lac*_::^*ss*^*dsbA-nlpD*^*(189–379)*^) were diluted in minimal M9-maltose medium and grown at 37°C. Mid-log cultures were then diluted into M9-maltose medium only or supplemented with 150μM (**A, D-E**) or 1mM (**B-C**) IPTG. Cells were further grown at 37°C to an OD_600_ of 0.2–0.3 before they were visualized on 2% agarose pads with DIC optics. Bar = 10μm.
(TIF)

S3 Fig OM localization of NlpD is required for proper cell separation. (**A**) Cells of MT50 (^*ΔSS*^*nlpD ΔenvC*), TB140 (*ΔenvC*), or MT50 (^*ΔSS*^*nlpD ΔenvC*) harboring the integrated expression constructs attHKMT20 (*P*_*lac*_::*nlpD*^*WT*^), attHKMT12 (*P*_*lac*_::*nlpD* ^*(S27D)*^), or attHKMT121 (*P*_*lac*_::^*ss*^*dsbA-nlpD* ^*(27–379)*^) were grown as indicated below in M9-maltose alone or supplemented with 150μM IPTG before harvesting for whole-cell extract preparation. Protein concentrations of the resulting extracts were normalized. The proteins were then separated by SDS-PAGE, transferred to PVDF, and NlpD was detected with the affinity-purified anti-NlpD antibody. The arrow indicates the NlpD variant present in each strain. The asterisk denotes a possible degradation product of NlpD^(27–379)^. (**B-E**) Overnight cultures of MT50 (^*ΔSS*^*nlpD ΔenvC*) harboring the integrated expression constructs (**B**) attHKMT20 (*P*_*lac*_::*nlpD*^*WT*^), (**D**) attHKMT12 (*P*_*lac*_::*nlpD* ^*(S27D)*^), or (**E**) attHKMT121 (*P*_*lac*_::^*ss*^*dsbA-nlpD* ^*(27–379)*^) were diluted in minimal M9-maltose medium and grown at 37°C until mid-log. Cultures were then diluted into M9-maltose only or with the indicated IPTG concentration. As a control, (**C**) TB140 (*ΔenvC*) were also grown under the same conditions in medium lacking IPTG. The cells were grown at 37°C to an OD_600_ of 0.25–0.35 and then visualized on 2% agarose pads with DIC optics. Bar = 10μm.
(TIF)

S4 Fig Assessing potential dominant negative effects and localization of NlpD variants with altered subcellular localization. (**A-C**) Cells of TB140 (*ΔenvC*) harboring the integrated expression constructs (**A**) attHKMT20 (*P*_*lac*_::*nlpD*^*WT*^), (**B**) attHKMT12 (*P*_*lac*_::*nlpD* ^*(S27D)*^), or (**C**) attHKMT121 (*P*_*lac*_::^*ss*^*dsbA-nlpD* ^*(27–379)*^) were grown overnight in LB with 50μM IPTG. The overnight cultures were diluted in minimal M9-maltose medium supplemented with 150μM IPTG. Cells were grown at 30°C to an OD_600_ of 0.1–0.2 before they were visualized on 2% agarose pads with DIC optics. (**D-E**) Overnight cultures of MT47 (^*ΔSS*^*nlpD*) harboring the integrated expression constructs (**D**) attHKNP20 (*P*_*lac*_::*nlpD*^*WT*^*-mCherry*), (**E**) attHKMT21 (*P*_*lac*_::*nlpD*^*S27D*^*-mCherry*), or (**F**) attHKMT147 (*P*_*lac*_::^*ss*^*dsbA-nlpD* ^*(27–379)*^*-mCherry*) were diluted in minimal M9-maltose medium supplemented with 50μM (**F**), 150μM (**D**), or 250μM (**E**) IPTG. Cells were grown at 30°C to an OD_600_ of 0.4 before they were visualized on 2% agarose pads by phase contrast and fluorescence microscopy. The subcellular localization of each NlpD variant is indicated in square brackets: OM, outer membrane; IM, inner membrane; peri, periplasm. Bar = 4μm.
(TIF)

S5 Fig Chaining phenotype of *ΔenvC ΔyraP* or *ΔenvC ΔtolQ-pal* mutants. Cells of (**A**) TB28 (WT), (**B**) TB140 (*ΔenvC*), (**C**) MT50 (*ΔenvC* ^*ΔSS*^*nlpD*), (**D**) MT135 (*ΔenvC ΔyraP*), and (**E**) MT55 (*ΔenvC ΔtolQ-pal*) were diluted in LB medium, grown at 30°C to an OD_600_ of 0.2–0.3, stained with the membrane dye FM 4-64FX (final concentration of 5 μg/ml) for 10 min before fixation. Stained cells were then visualized by phase contrast and fluorescence microscopy. Bar = 10μm.
(TIF)

S6 Fig Phylogenetic distribution of NlpD and YraP. A phylogenetic tree of representative bacterial species is presented. The distributions of NlpD (blue) and YraP (magenta) are plotted as bars over the tree. To identify species with occurrence of these proteins, *E*. *coli* YraP and NlpD sequences were used to search the NCBI nr database for homologs with BLASTP program, applying an empirically determined E-value cutoff of 1.00 x 10^−20^.
(TIF)

S7 Fig The different YraP variants are in the proper subcellular location and expressed to produce similar protein levels. Overnight cultures of MT140 (*ΔyraP*) harboring the integrated expression constructs (**A**) attλMT197 (*P*_*lac*_::*yraP*^*WT*^*-mCherry*), (**B**) attλMT199 (*P*_*lac*_::*yraP*^*IM*^*-mCherry*), or (**C**) attλMT210 (*P*_*lac*_::^*ss*^*dsbA-yraP*^*peri*^*-mCherry*) were diluted in minimal M9-maltose medium supplemented with 250μM IPTG. Cells were grown at 30°C to an OD_600_ of 0.2, washed, osmotically shocked, and visualized by phase contrast and fluorescence microscopy. Arrows indicate peripheral OM mCherry signals (**A**), signals that track with the inner membrane in (**B**), or fill the increased periplasmic spaces in plasmolysis bays (**C**). Bar = 4μm. (**D**) Cells expressing no mCherry protein (TB143) or MT140 (*ΔyraP*) cells expressing different YraP-mCherry fusions from the integrated constructs attλMT197 (*P*_*lac*_::*yraP*^*WT*^*-mCherry*), attλMT199 (*P*_*lac*_::*yraP*^*IM*^*-mCherry*), or attλMT210 (*P*_*lac*_::^*ss*^*dsbA-yraP*^*peri*^*-mCherry*) were diluted in minimal M9-maltose medium supplemented with the indicated concentration of IPTG, grown at 30°C, and then harvested for whole-cell extract preparation. Protein concentrations of the resulting extracts were normalized. The proteins were then separated by SDS-PAGE, transferred to PVDF, and the mCherry fusion was detected with anti-mCherry antibody. The arrow indicates the YraP variant present in each strain.
(TIF)

S8 Fig Localization of YraP or NlpD in cells blocked for division by SulA. Overnight cultures of (**A-B**) MT140 (*ΔyraP*) harboring pMT224 (P_*ara*_::*sulA*) and the integrated construct attλMT197 (*P*_*lac*_::*yraP-mCherry*) or (**C-D**) MT47 (^*ΔSS*^*nlpD*) harboring pMT224 and the integrated expression construct attHKNP20 (*P*_*lac*_::*nlpD-mCherry*) were diluted in minimal M9-maltose medium supplemented with either 25μM (**A-B**) or 100μM (**C-D**) IPTG and grown at 30°C until mid-log. Cultures were then backdiluted into M9-maltose medium with the indicated IPTG concentration with or without 0.2% arabinose to induce the production of the FtsZ antagonist SulA, as indicated. Cells were grown at 30°C to an OD_600_ of 0.2 before they were visualized on 2% agarose pads by phase contrast and fluorescence microscopy. Arrows indicate localization of the protein fusion to division sites. Bar = 10μm.
(TIF)

S1 Table List of strains used in this study. (PDF)

S2 Table List of plasmids used in this study. (PDF)

S1 Text Details for plasmid constructions for this study. (PDF)
