# Supplementary figures and images for: NlpD links cell wall remodeling and outer membrane invagination during cytokinesis in Escherichia coli
Source: PLoS Genet. 2017 Jul 14;13(7):e1006888. doi: 10.1371/journal.pgen.1006888 (PMC5533458; doi:10.1371/journal.pgen.1006888)

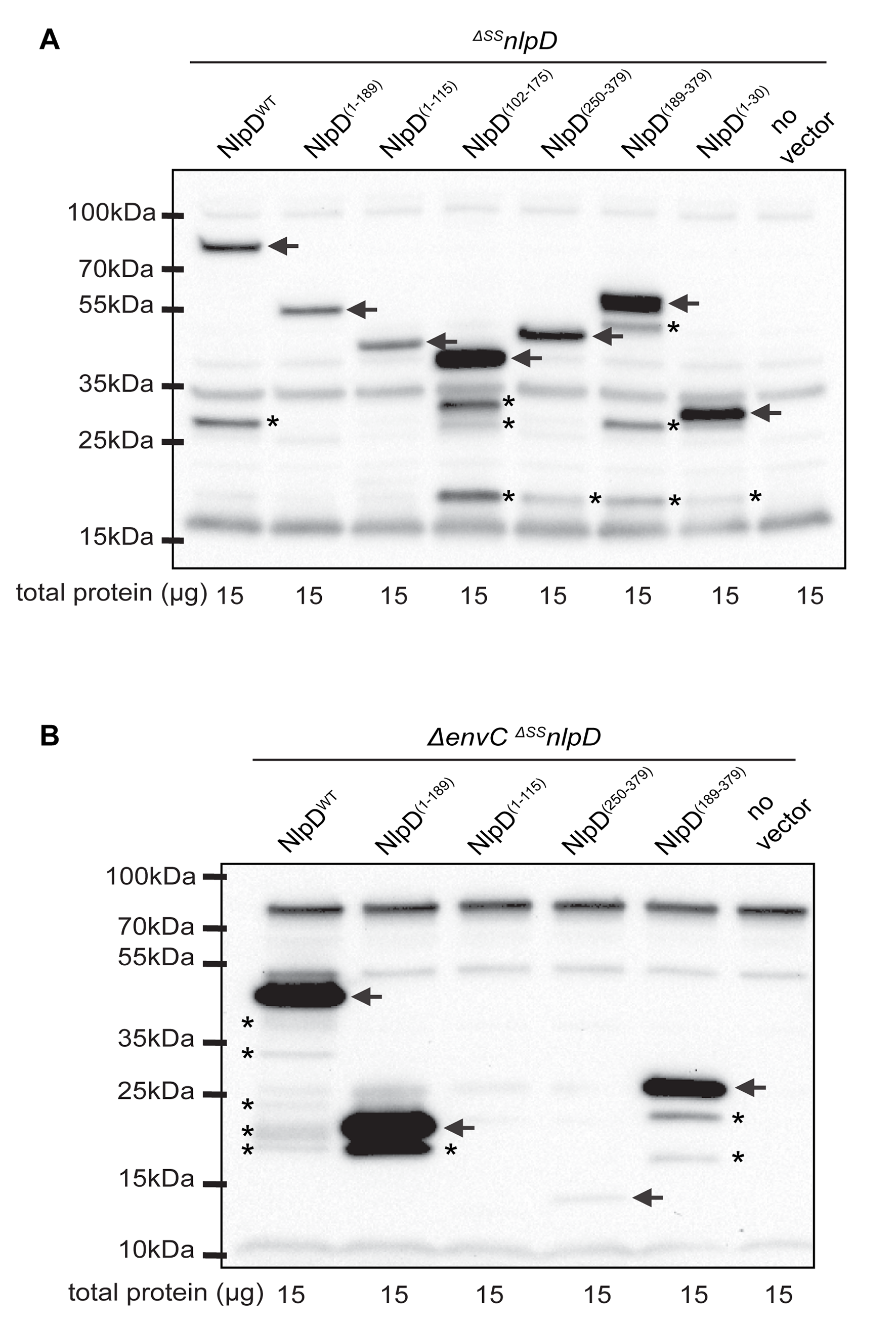

Supplement: S1 Fig — (A) Cells of MT47 (ΔSSnlpD) alone or expressing different NlpD-mCherry fusions from the integrated constructs attHKNP20 (Plac::nlpDWT-mCherry), attHKMT101 (Plac::nlpD(1–189)-mCherry), attHKMT103 (Plac::nlpD(1–115)-mCherry), attHKMT178 (Plac::ssdsbA-nlpD(102–175)-mCherry), attHKMT180 (Plac::ssdsbA-nlpD(250–379)-mCherry), attHKMT182 (Plac::ssdsbA-nlpD(189–379)-mCherry), or attHKMT149 (Plac::nlpD(1–30)-mCherry) were diluted in minimal M9-maltose medium supplemented with IPTG at concentrations indicated in Fig 2, grown at 30°C, and then harvested for whole-cell extract preparation. (B) Cells of MT50 (ΔSSnlpD ΔenvC) alone or expressing different NlpD variants from the integrated constructs attHKMT20 (Plac::nlpDWT), attHKMT102 (Plac::nlpD(1–189)), attHKMT104 (Plac::nlpD(1–115)), attHKMT179 (Plac::ssdsbA-nlpD(250–379)), or attHKMT181 (Plac::ssdsbA-nlpD(189–379)) were diluted in minimal M9-maltose medium alone (MT50) or supplemented with either 150μM (attHKMT20, attHKMT179, or attHKMT181) or 1mM (attHKMT102 or attHKMT104) IPTG, grown at 37°C, and then harvested for whole-cell extract preparation. Proteins in the resulting extracts were separated by SDS-PAGE, transferred to PVDF, and NlpD was detected with anti-mCherry (A) or affinity-purified anti-NlpD (B) antibodies. The arrows indicate the NlpD variant present in each strain. The asterisks denote possible degradation products of each variant. (TIF) [file pgen.1006888.s001.tif]

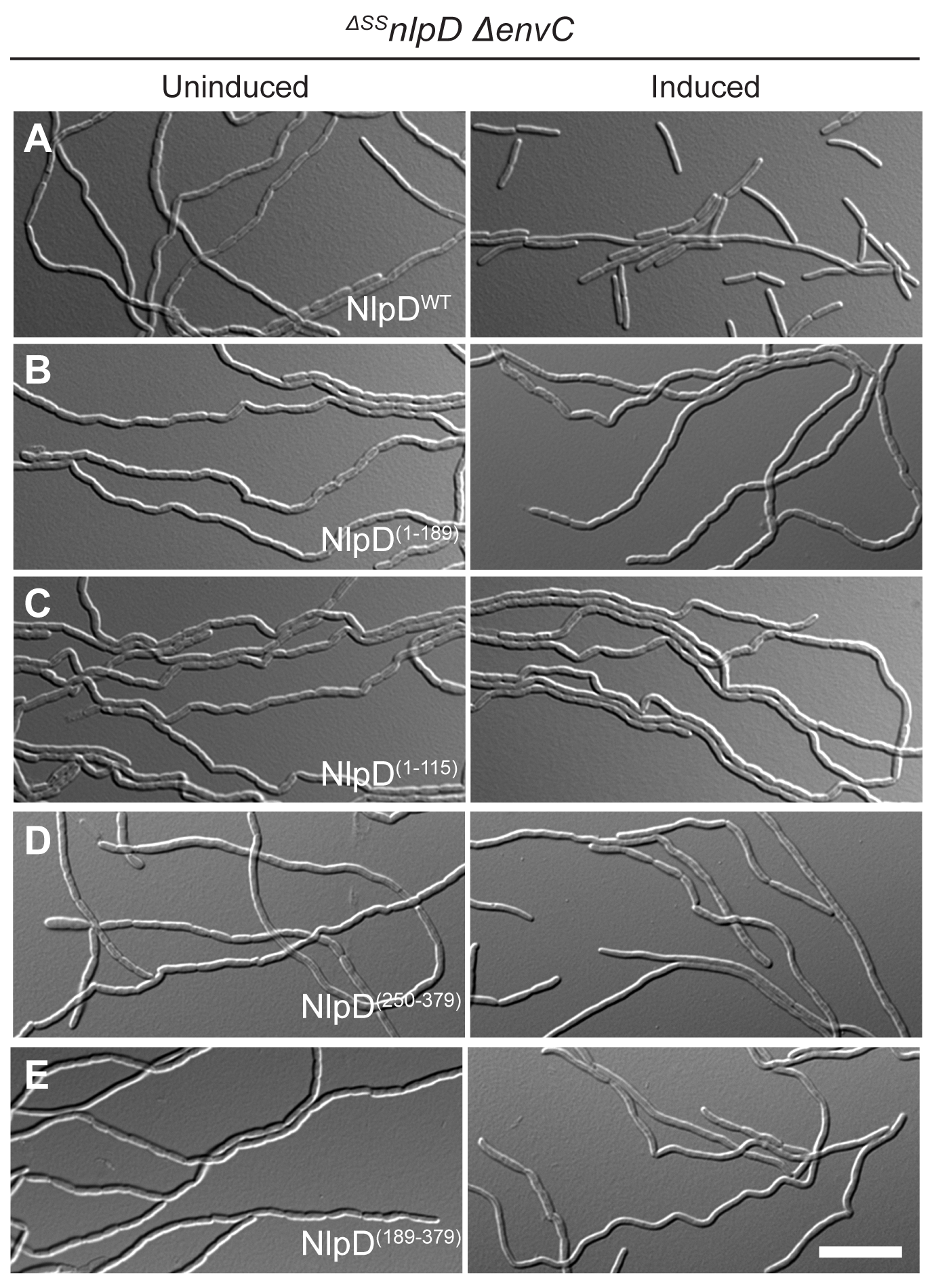

Supplement: S2 Fig — (A-E) Overnight cultures of MT50 harboring the integrated expression constructs (A) attHKMT20 (Plac::nlpDWT), (B) attHKMT102 (Plac::nlpD(1–189)), (C) attHKMT104 (Plac::nlpD(1–115)), (D) attHKMT179 (Plac::ssdsbA-nlpD(250–379)), or (E) attHKMT181 (Plac::ssdsbA-nlpD(189–379)) were diluted in minimal M9-maltose medium and grown at 37°C. Mid-log cultures were then diluted into M9-maltose medium only or supplemented with 150μM (A, D-E) or 1mM (B-C) IPTG. Cells were further grown at 37°C to an OD600 of 0.2–0.3 before they were visualized on 2% agarose pads with DIC optics. Bar = 10μm. (TIF) [file pgen.1006888.s002.tif]

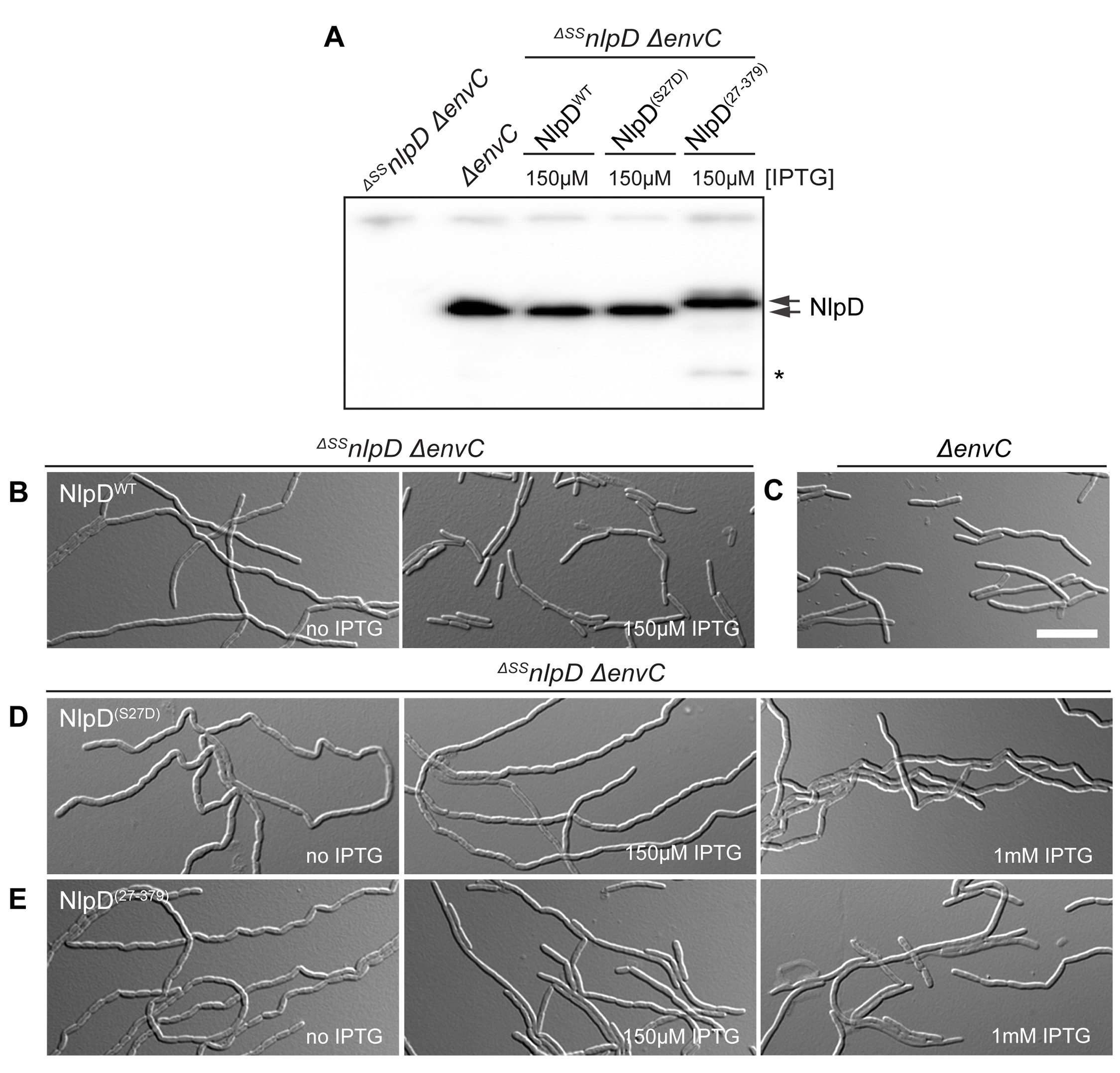

Supplement: S3 Fig — (A) Cells of MT50 (ΔSSnlpD ΔenvC), TB140 (ΔenvC), or MT50 (ΔSSnlpD ΔenvC) harboring the integrated expression constructs attHKMT20 (Plac::nlpDWT), attHKMT12 (Plac::nlpD (S27D)), or attHKMT121 (Plac::ssdsbA-nlpD (27–379)) were grown as indicated below in M9-maltose alone or supplemented with 150μM IPTG before harvesting for whole-cell extract preparation. Protein concentrations of the resulting extracts were normalized. The proteins were then separated by SDS-PAGE, transferred to PVDF, and NlpD was detected with the affinity-purified anti-NlpD antibody. The arrow indicates the NlpD variant present in each strain. The asterisk denotes a possible degradation product of NlpD(27–379). (B-E) Overnight cultures of MT50 (ΔSSnlpD ΔenvC) harboring the integrated expression constructs (B) attHKMT20 (Plac::nlpDWT), (D) attHKMT12 (Plac::nlpD (S27D)), or (E) attHKMT121 (Plac::ssdsbA-nlpD (27–379)) were diluted in minimal M9-maltose medium and grown at 37°C until mid-log. Cultures were then diluted into M9-maltose only or with the indicated IPTG concentration. As a control, (C) TB140 (ΔenvC) were also grown under the same conditions in medium lacking IPTG. The cells were grown at 37°C to an OD600 of 0.25–0.35 and then visualized on 2% agarose pads with DIC optics. Bar = 10μm. (TIF) [file pgen.1006888.s003.tif]

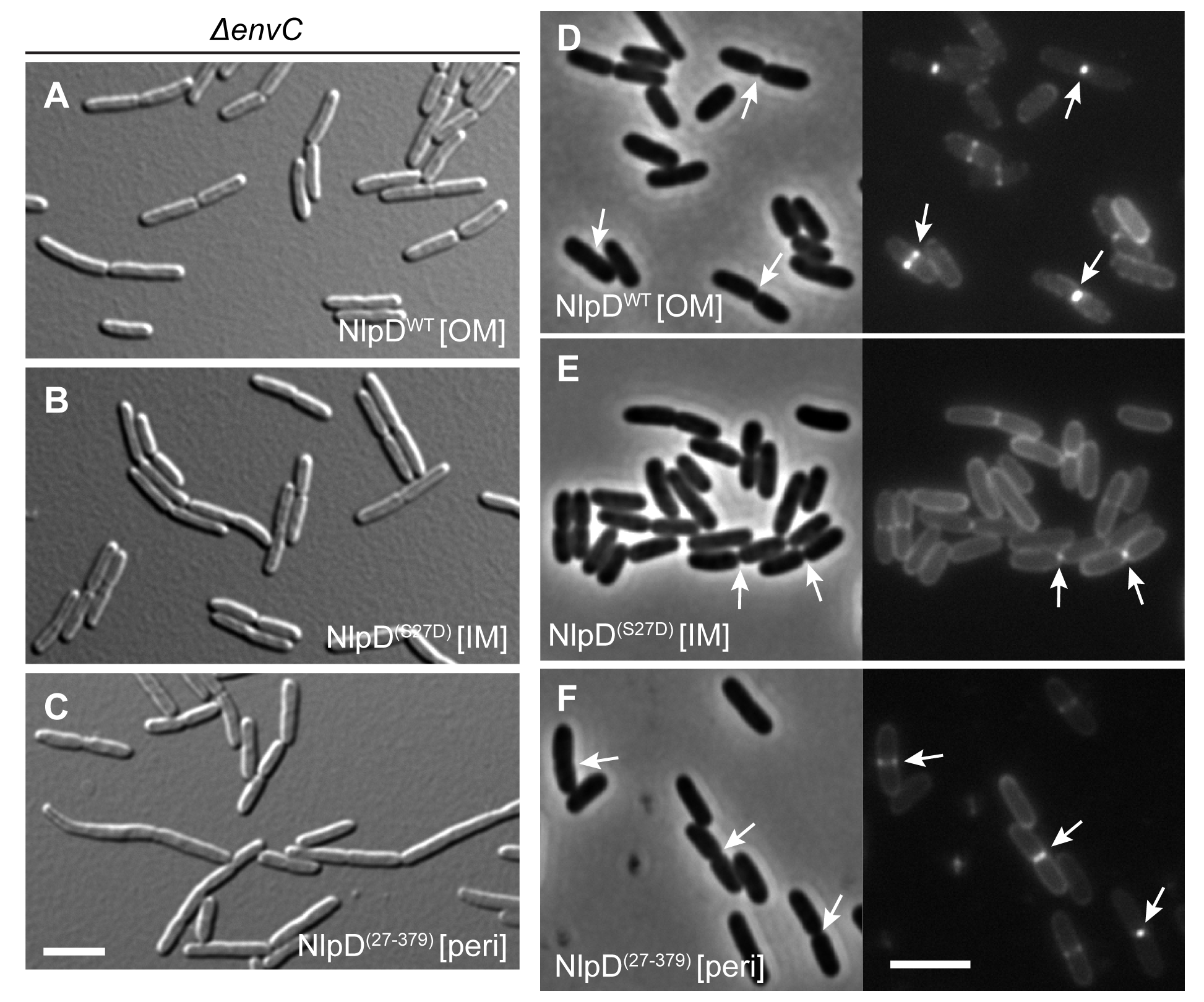

Supplement: S4 Fig — (A-C) Cells of TB140 (ΔenvC) harboring the integrated expression constructs (A) attHKMT20 (Plac::nlpDWT), (B) attHKMT12 (Plac::nlpD (S27D)), or (C) attHKMT121 (Plac::ssdsbA-nlpD (27–379)) were grown overnight in LB with 50μM IPTG. The overnight cultures were diluted in minimal M9-maltose medium supplemented with 150μM IPTG. Cells were grown at 30°C to an OD600 of 0.1–0.2 before they were visualized on 2% agarose pads with DIC optics. (D-E) Overnight cultures of MT47 (ΔSSnlpD) harboring the integrated expression constructs (D) attHKNP20 (Plac::nlpDWT-mCherry), (E) attHKMT21 (Plac::nlpDS27D-mCherry), or (F) attHKMT147 (Plac::ssdsbA-nlpD (27–379)-mCherry) were diluted in minimal M9-maltose medium supplemented with 50μM (F), 150μM (D), or 250μM (E) IPTG. Cells were grown at 30°C to an OD600 of 0.4 before they were visualized on 2% agarose pads by phase contrast and fluorescence microscopy. The subcellular localization of each NlpD variant is indicated in square brackets: OM, outer membrane; IM, inner membrane; peri, periplasm. Bar = 4μm. (TIF) [file pgen.1006888.s004.tif]

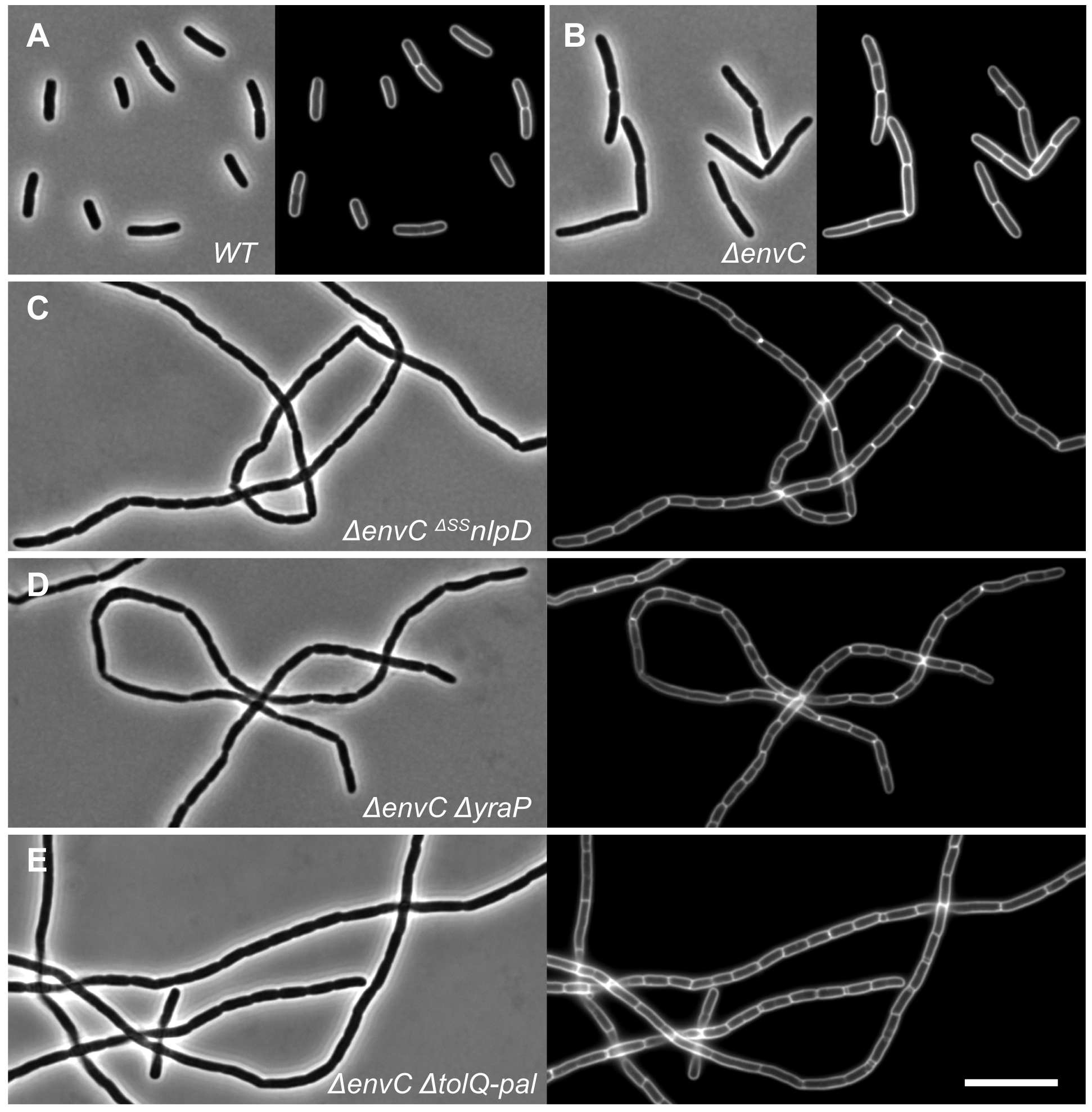

Supplement: S5 Fig — Cells of (A) TB28 (WT), (B) TB140 (ΔenvC), (C) MT50 (ΔenvC ΔSSnlpD), (D) MT135 (ΔenvC ΔyraP), and (E) MT55 (ΔenvC ΔtolQ-pal) were diluted in LB medium, grown at 30°C to an OD600 of 0.2–0.3, stained with the membrane dye FM 4-64FX (final concentration of 5 μg/ml) for 10 min before fixation. Stained cells were then visualized by phase contrast and fluorescence microscopy. Bar = 10μm. (TIF) [file pgen.1006888.s005.tif]

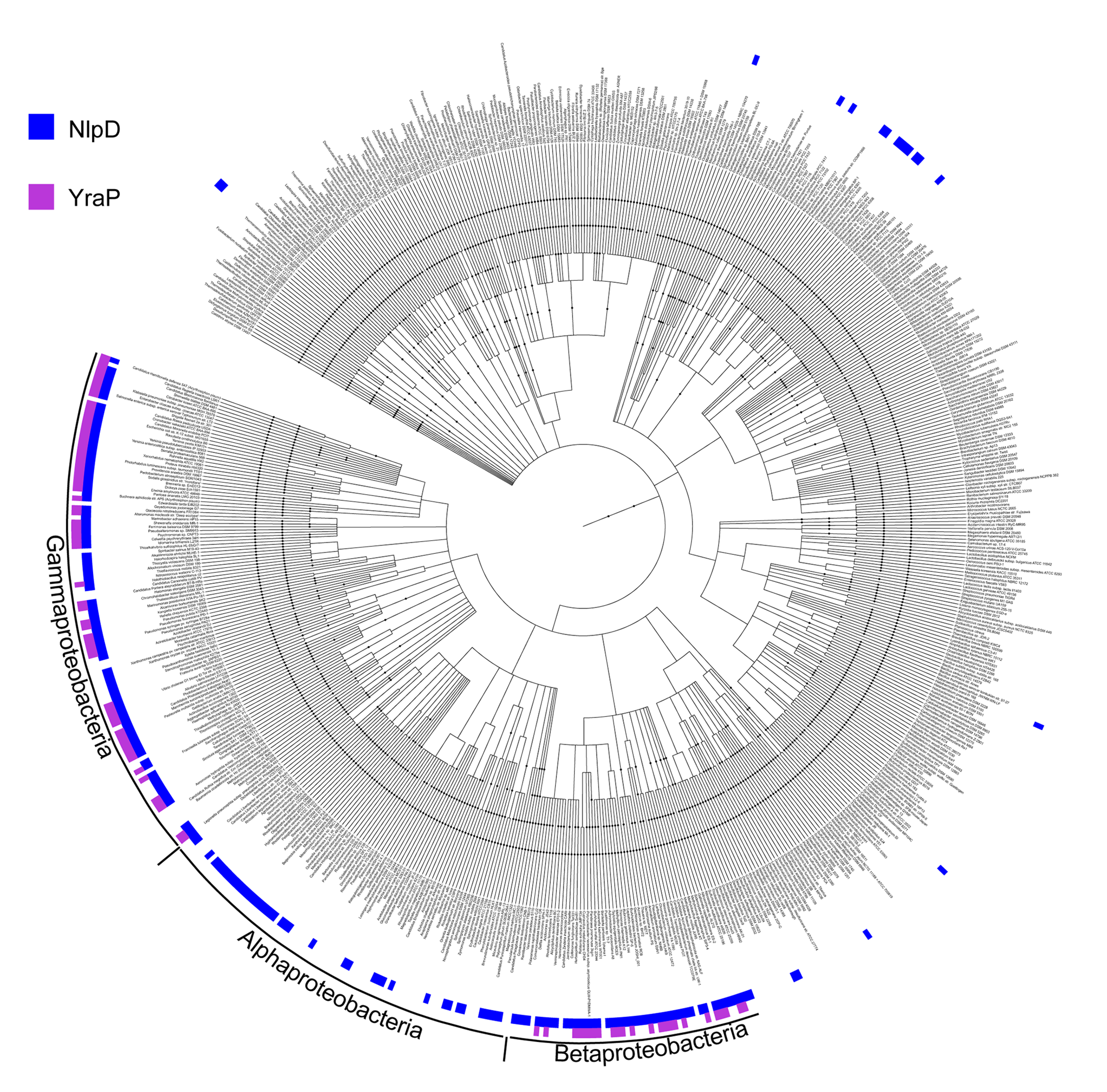

Supplement: S6 Fig — A phylogenetic tree of representative bacterial species is presented. The distributions of NlpD (blue) and YraP (magenta) are plotted as bars over the tree. To identify species with occurrence of these proteins, E. coli YraP and NlpD sequences were used to search the NCBI nr database for homologs with BLASTP program, applying an empirically determined E-value cutoff of 1.00 x 10^−20^. (TIF) [file pgen.1006888.s006.tif]

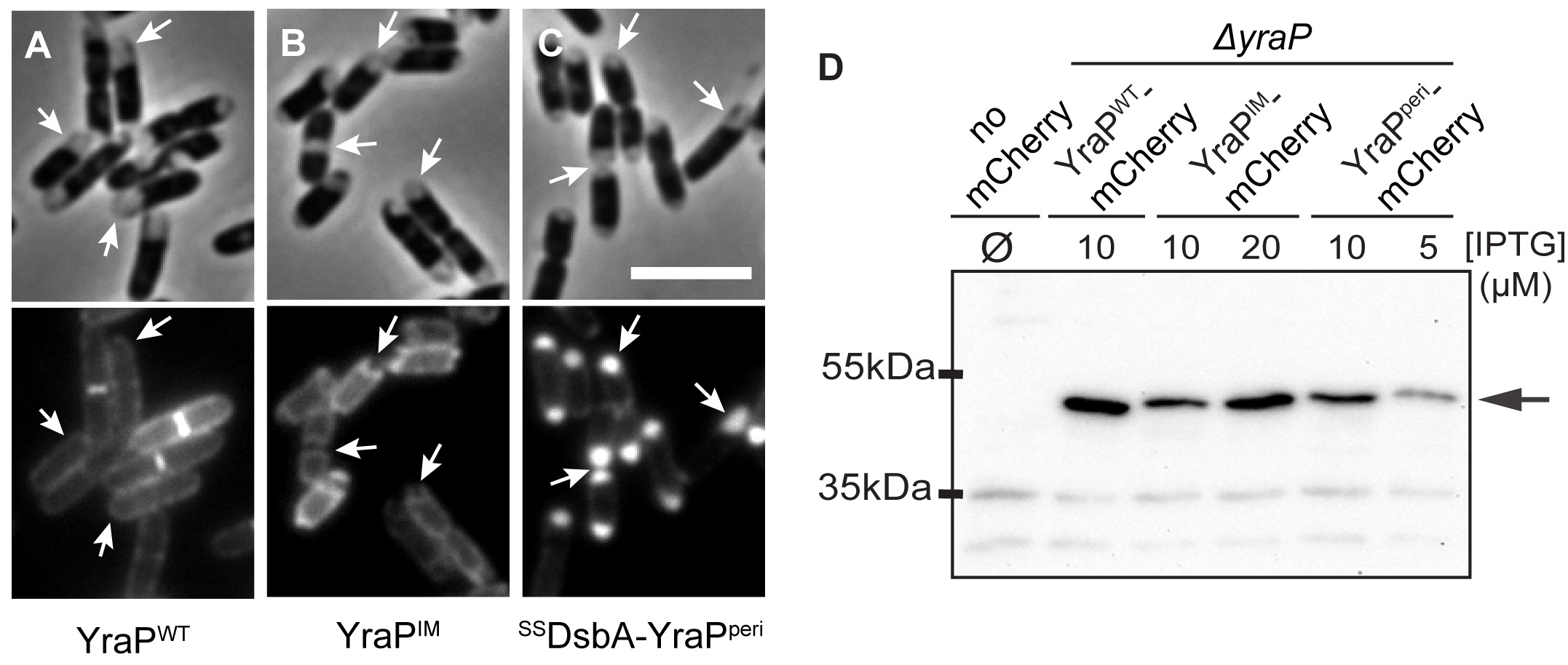

Supplement: S7 Fig — Overnight cultures of MT140 (ΔyraP) harboring the integrated expression constructs (A) attλMT197 (Plac::yraPWT-mCherry), (B) attλMT199 (Plac::yraPIM-mCherry), or (C) attλMT210 (Plac::ssdsbA-yraPperi-mCherry) were diluted in minimal M9-maltose medium supplemented with 250μM IPTG. Cells were grown at 30°C to an OD600 of 0.2, washed, osmotically shocked, and visualized by phase contrast and fluorescence microscopy. Arrows indicate peripheral OM mCherry signals (A), signals that track with the inner membrane in (B), or fill the increased periplasmic spaces in plasmolysis bays (C). Bar = 4μm. (D) Cells expressing no mCherry protein (TB143) or MT140 (ΔyraP) cells expressing different YraP-mCherry fusions from the integrated constructs attλMT197 (Plac::yraPWT-mCherry), attλMT199 (Plac::yraPIM-mCherry), or attλMT210 (Plac::ssdsbA-yraPperi-mCherry) were diluted in minimal M9-maltose medium supplemented with the indicated concentration of IPTG, grown at 30°C, and then harvested for whole-cell extract preparation. Protein concentrations of the resulting extracts were normalized. The proteins were then separated by SDS-PAGE, transferred to PVDF, and the mCherry fusion was detected with anti-mCherry antibody. The arrow indicates the YraP variant present in each strain. (TIF) [file pgen.1006888.s007.tif]

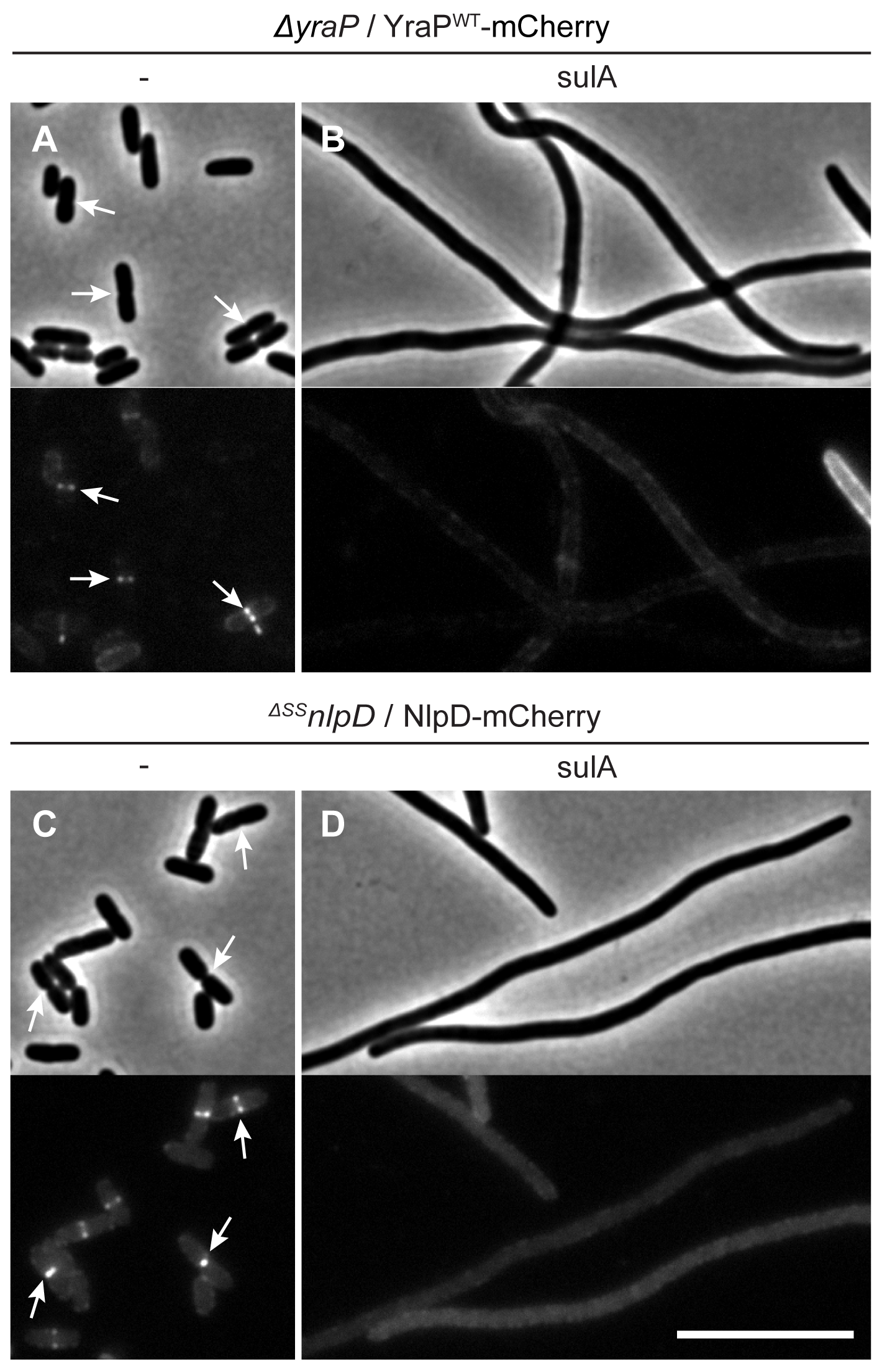

Supplement: S8 Fig — Overnight cultures of (A-B) MT140 (ΔyraP) harboring pMT224 (Para::sulA) and the integrated construct attλMT197 (Plac::yraP-mCherry) or (C-D) MT47 (ΔSSnlpD) harboring pMT224 and the integrated expression construct attHKNP20 (Plac::nlpD-mCherry) were diluted in minimal M9-maltose medium supplemented with either 25μM (A-B) or 100μM (C-D) IPTG and grown at 30°C until mid-log. Cultures were then backdiluted into M9-maltose medium with the indicated IPTG concentration with or without 0.2% arabinose to induce the production of the FtsZ antagonist SulA, as indicated. Cells were grown at 30°C to an OD600 of 0.2 before they were visualized on 2% agarose pads by phase contrast and fluorescence microscopy. Arrows indicate localization of the protein fusion to division sites. Bar = 10μm. (TIF) [file pgen.1006888.s008.tif]
