# Supplementary material for: NlpD links cell wall remodeling and outer membrane invagination during cytokinesis in Escherichia coli
Source: PLoS Genet. 2017 Jul 14;13(7):e1006888. doi: 10.1371/journal.pgen.1006888 (PMC5533458; doi:10.1371/journal.pgen.1006888)
Supplement: S1 Table — (PDF) [file pgen.1006888.s009.pdf]

S1 Table. Strains used in this study

Strain	Genotype[a]	Source/Reference[b]
DH5α	*F– hsdR17 deoR recA1 endA1 phoA supE44 thi-1 gyrA96 relA1 Δ(lacZYA-argF)U169 ϕ80dlacZΔM15*	Gibco BRL
BL21(λDE3)	*ompT rB– mB– (PlacUV5::T7gene1)*	Novagen
MG1655	*rph-1 ilvG rfb-50*	[1]
AAY22	TB28 *ΔamiC::frt ΔyraP::Kan*^{R}	P1(MT129) x TB143
MT46	TB28 $^{\Delta SS}$*nlpD::Kan*^{R}	P1(λRed) x TB28
MT47	TB28 $^{\Delta SS}$*nlpD::frt*	MT46/pCP20
MT50	TB28 $^{\Delta SS}$*nlpD::frt ΔenvC::Kan*^{R}	P1(TB134) x MT47
MT51	TB28 *ΔtolQ-pal::Kan*^{R}	P1(λRed) x TB28
MT53	TB28 $^{\Delta SS}$*nlpD::frt ΔtolQ-pal::Kan*^{R}	P1(λRed) x MT47
MT55	TB28 *ΔenvC::frt ΔtolQ-pal::Kan*^{R}	P1(λRed) x TB140
MT56	TB28 *ΔamiC::frt ΔtolQ-pal::Kan*^{R}	P1(MT51) x TB143
MT92	TB28 $^{\Delta SS}$*nlpD::frt ΔtolQ-pal::frt*	MT53/pCP20
MT96	TB28 $^{\Delta SS}$*nlpD::frt ΔtolQ-pal::frt ΔenvC::Kan*^{R}	P1(TB134) x MT92
MT121	MG1655 $^{\Delta SS}$*nlpD::Kan*^{R}	P1(MT46) x MG1655
MT122	MG1655 $^{\Delta SS}$*nlpD::frt*	MT121/pCP20
MT123	MG1655 $^{\Delta SS}$*nlpD::frt ΔamiC::Kan*^{R}	P1(TB137) x MT122
MT129	TB10 *ΔyraP::Kan*^{R}	λRed
MT135	TB28 *ΔenvC::frt ΔyraP::Kan*^{R}	P1(MT129) x TB140
MT140	TB28 *ΔyraP::Kan*^{R}	P1(MT129) x TB28
MT141	TB28 $^{\Delta SS}$*nlpD::frt ΔyraP::Kan*^{R}	P1(MT129) x MT47
MT147	TB28 $^{\Delta SS}$*nlpD::frt ΔtolQ-pal::frt ΔyraP::Kan*^{R}	P1(MT129) x MT92
MT149	TB28 *ΔamiC::frt ΔtolQ-pal::Kan*^{R}	P1(MT51) x TB143
MT150	TB28 *ΔamiC::frt ΔyraP::Kan*^{R}	P1(MT129) x TB143
MT174	TB28 *ΔamiC::frt ΔtolQ-pal::frt*	MT56/pCP20

Strain	Genotype[a]	Source/Reference[b]
MT176	TB28 $^{\Delta SS}$*nlpD::frt ΔamiC::Kan*^{R}	P1(TB137) x MT47
MT177	TB28 $^{\Delta SS}$*nlpD::frt ΔyraP::frt*	MT141/pCP20
MT178	TB28 *ΔamiC::frt ΔtolQ-pal::frt ΔyraP::Kan*^{R}	P1(MT129) x MT174
MT179	TB28 $^{\Delta SS}$*nlpD::frt ΔyraP::frt ΔenvC::Kan*^{R}	P1(TB134) x MT177
TB10	MG1655 *λΔcro-bio nad::Tn10*	[2]
TB28	MG1655 *ΔlacIZYA*::*frt*	[3]
TB134	TB28 *ΔenvC::Kan*^{R}	[4]
TB137	TB28 *ΔamiC::Kan*^{R}	[4]
TB140	TB28 *ΔenvC::frt*	[4]
TB143	TB28 *ΔamiC::frt*	[5]

[a] The Kan^{R} cassette is flanked by *frt* sites for removal by FLP recombinase. An *frt* scar remains following removal of the cassette using FLP recombinase expressed from pCP20.

[b] Strain constructions by P1 transduction are described using the shorthand: P1(donor) x recipient. Transductants were selected on LB Kan, LB Tet, or LB Cm plates where appropriate. λRed indicates strains constructed by recombineering (see Experimental Procedures for details). Strains resulting from the removal of a drug resistance cassette using pCP20 are indicated as: Parental strain/pCP20.
