# Supplementary material for: NlpD links cell wall remodeling and outer membrane invagination during cytokinesis in Escherichia coli
Source: PLoS Genet. 2017 Jul 14;13(7):e1006888. doi: 10.1371/journal.pgen.1006888 (PMC5533458; doi:10.1371/journal.pgen.1006888)
Supplement: S2 Table — (PDF) [file pgen.1006888.s010.pdf]

S2 Table. Plasmids used in this study

Plasmid	Genotype[a]	Origin	Source or Reference
pBAD24	*bla araC* P_{ara}*::empty*	pBR/ColE1	[6]
pINT-ts	*bla repA(ts) cI857(ts)* $P_{\lambda R}$*::int(λ)*	R6K	[7]
pKD46	*bla repA101(ts) araC* P_{ara}*::γ-β-exo*	pSC101	[8]
pMT12	*att*HK022 *tetA tetR lacI* P_{lac}*::nlpD*$^{(S27D)}$	R6K	This study
pMT20	*att*HK022 *tetA tetR lacI* P_{lac}*::nlpD*$^{(1-379)}$	R6K	[9]
pMT21	*att*HK022 *tetA tetR lacI* P_{lac}*::nlpD*$^{(S27D)}$*-mCherry*	R6K	This study
pMT94	*cat lacI* P_{lac}*::nativeRBS_ybgC_tolQRA*	pACYC/p15A	This study
pMT101	*att*HK022 *tetA tetR lacI* P_{lac}*::nlpD*$^{(1-189)}$*-mCherry*	R6K	This study
pMT102	*att*HK022 *tetA tetR lacI* P_{lac}*::nlpD*$^{(1-189)}$	R6K	This study
pMT103	*att*HK022 *tetA tetR lacI* P_{lac}*::nlpD*$^{(1-115)}$*-mCherry*	R6K	This study
pMT104	*att*HK022 *tetA tetR lacI* P_{lac}*::nlpD*$^{(1-115)}$	R6K	This study
pMT121	*att*HK022 *tetA tetR lacI* P_{lac}*::* ^{ss}*dsbA-nlpD*$^{(27-379)}$	R6K	This study
pMT147	*att*HK022 *tetA tetR lacI* P_{lac}*::* ^{ss}*dsbA-nlpD*$^{(27-379)}$*-mCherry*	R6K	This study
pMT149	*att*HK022 *tetA tetR lacI* P_{lac}*::nlpD*$^{(1-30)}$*-mCherry*	R6K	This study
pMT178	*att*HK022 *tetA tetR lacI* P_{lac}*::* ^{ss}*dsbA-nlpD*$^{(102-175)}$*-mCherry*	R6K	This study
pMT179	*att*HK022 *tetA tetR lacI* P_{lac}*::* ^{ss}*dsbA-nlpD*$^{(250-379)}$	R6K	This study
pMT180	*att*HK022 *tetA tetR lacI* P_{lac}*::* ^{ss}*dsbA-nlpD*$^{(250-379)}$*-mCherry*	R6K	This study
pMT181	*att*HK022 *tetA tetR lacI* P_{lac}*::* ^{ss}*dsbA-nlpD*$^{(189-379)}$	R6K	This study
pMT182	*att*HK022 *tetA tetR lacI* P_{lac}*::* ^{ss}*dsbA-nlpD*$^{(189-379)}$*-mCherry*	R6K	This study
pMT187	*aadA repA(ts) lacI*^{q} P_{lac}*::envC-LE* <u>SceI</u> *cI857* $P_{\lambda R}$*::i-sceI*	pSC101	This study
pMT196	*attλ cat lacI* P_{lac}*::yraP*$^{(1-191)}$	R6K	This study
pMT197	*attλ cat lacI* P_{lac}*::yraP*$^{(1-191)}$*-mCherry*	R6K	This study

Plasmid	Genotype[a]	Origin	Source or Reference
pMT198	*attλ cat lacI* P_{lac}*::yraP*$^{(VA\text{-}20,21\text{-}DE)}$	R6K	This study
pMT199	*attλ cat lacI* P_{lac}*::yraP*$^{(VA\text{-}20,21\text{-}DE)}$*-mCherry*	R6K	This study
pMT209	*attλ cat lacI* P_{lac}*::* ^{ss}*dsbA-yraP*$^{(24\text{-}191)}$	R6K	This study
pMT210	*attλ cat lacI* P_{lac}*::* ^{ss}*dsbA-yraP*$^{(24\text{-}191)}$*-mCherry*	R6K	This study
pMT224	*bla araC* P_{ara}*::sulA*	pBR/ColE1	This study
pNP16	*attHK022 tetA tetR lacI*^{q} $P_{lac\text{-}m3}$*::amiC-GFP*	R6K	[5]
pNP20	*att*HK022 *tetA tetR lacI*^{q} P_{lac}*::nlpD*$^{(1\text{-}379)}$*-mCherry*	R6K	[5]
pTB102	*cat repA(ts) cI857(ts)* $P_{\lambda R}$*::int(HK022)*	pSC101	[10]
pTU119	*bla lacI*^{q} P_{T7}*::h-sumo-nlpD*$^{(27\text{-}379)}$	pBR/ColE1	[11]

[a] P_{ara}, $P_{\lambda R}$, and P_{lac} indicate the arabinose, λR, and lactose promoters, respectively. $P_{lac\text{-}m3}$ is a *lac* promoter derivative with TATATT as its -10 element. ^{ss}*dsbA* corresponds to the first 24 codons of *dsbA* encoding its export signal to the periplasm. SceI indicates the presence of a substrate site for the I-SceI homing endonuclease. Numbers in superscript parenthesis indicate the amino acids included in the relevant clones. Unless indicated, the ribosome binding site (RBS) used for all constructs is the strong RBS of phage T7 *Φ*10 gene. Additionally, all mCherry fusion proteins contain a LEGPAGL peptide linker between the NlpD variant and mCherry protein.
