# Supplementary material for: NlpD links cell wall remodeling and outer membrane invagination during cytokinesis in Escherichia coli
Source: PLoS Genet. 2017 Jul 14;13(7):e1006888. doi: 10.1371/journal.pgen.1006888 (PMC5533458; doi:10.1371/journal.pgen.1006888)
Supplement: S1 Text — (PDF) [file pgen.1006888.s011.pdf]

Supporting material for:

NlpD links cell wall remodeling and outer membrane invagination during cytokinesis in *Escherichia coli*

Authors: Mary-Jane Tsang[1], Anastasiya A. Yakhnina[1] and Thomas G. Bernhardt[1*]

Affiliations:
[1]Department of Microbiology and Immunobiology
Harvard Medical School
Boston, MA 02115

*To whom correspondence should be addressed.
Thomas G. Bernhardt, Ph.D.
Harvard Medical School
Department of Microbiology and Immunobiology
Boston, Massachusetts 02115
e-mail: thomas_bernhardt@hms.harvard.edu

Plasmid Construction

Plasmids used in this study are listed below. PCR was performed using KOD polymerase (Novagen) for cloning purposes and *Taq* DNA polymerase (NEB) for diagnostic purposes, both according to the manufacturer's instructions. Unless otherwise indicated, MG1655 chromosomal DNA was used as the template. Restriction sites for use in plasmid constructions are bold, italicized and underlined in the primer sequences given below. Plasmid DNA and PCR fragments were purified using the Zyppy plasmid miniprep kit (Zymo Research) or the Qiaquick PCR purification kit (Qiagen), respectively.

<u>pMT12</u>

The plasmid pMT12 [*att*HK022 *tetA tetR lacI* P_{lac}::*nlpD*$^{(S27D)}$] was constructed as follows. The primers 5'-GACTCTCTTCCGGGCGCTATC-3'/5'-GTGCCGGTGGATTTGAAGTGTCATCACAGCCTGC-3' and 5'-GTTTCGCTATGGCTGGCAGGCTGTGATGACACTTC-3'/5'-GACGAAAGTGATTGCGCCTACC-3' were used in overlap extension PCR to amplify the *nlpD* gene from pTD23 and to perform site directed mutagenesis to create the S27D mutation in the *nlpD* gene. The resulting PCR product was purified, digested with XbaI and HindIII and ligated with similarly digested pNP20 [*att*HK022 *tetA tetR lacI*^{q} P_{lac}::*nlpD-mCherry*] [5].

<u>pMT18</u>

The plasmid pMT18 [*att*HK022 *bla lacI^q^* P_{lac}*::^ss^dsbA-nlpD^(27-379)^*] was constructed as follows. The primers 5'-GTCA***<u>GGATCC</u>***TCTGACACTTCAAATCCACCGGC-3' and 5'-GTCA***<u>AAGCTT</u>***CCGCCGATTTATCGCTGC-3' were used to amplify *nlpD(27-379)* from genomic DNA. The resulting PCR product was purified, digested with XbaI and XhoI and ligated with similarly digested pTB282 [*att*HK022 *bla lacI^q^* P_{lac}*::^ss^dsbA-sfGFP*] [5].

<u>pMT21</u>

The plasmid pMT21 [*att*HK022 *tetA tetR lacI* P_{lac}*::nlpD^(S27D)^-mCherry*] was constructed as follows. The primers 5'-TTAGGCACCCCAGGCTTTACAC-3' and 5'-GTCACTCGAGTCGCTGCGGCAAATAACGC-3' were used to amplify *nlpD(S27D)* from pMT12. The resulting PCR product was purified, digested with XbaI and XhoI and ligated with similarly digested pNP18 [*att*HK022 *tetA tetR lacI^q^* P_{lac}*::envC-mCherry*] [5].

<u>pMT94</u>

The plasmid pMT94 [*cat lacI* P_{lac}*::nativeRBS_ybgC_tolQRA*] was constructed as follows and involved several steps to assemble. First, the *lacI^q^*/P_{lac} containing BglII/XbaI fragment of pHC528 [*attλ cat lacI^q^* P_{lac}*::slmA*] [12] was used to replace the corresponding fragment of pDY31 [*cat* P_{ara}*::nativeRBS-amiB*] [13]. The primers 5'-GTCA***<u>TCTAGA</u>***CTCTAACTTTTGTTGCATTACCGGGATG-3' and 5'-GTCA***<u>ATCGAT</u>***TTACGGTTTGAAGTCCAATGGCG-3' were used to amplify *nativeRBS_ybgC_tolQRA* from genomic DNA. The resulting PCR product was purified, digested with XbaI and ClaI and ligated into the above plasmid digested with the same enzymes to create pMT94.

pMT101

The plasmid pMT101 [*att*HK022 *tetA tetR lacI* P_{lac}::*nlpD*$^{(1-189)}$-*mCherry*] was constructed as follows. The primers 5'-TTAGGCACCCCAGGCTTTACAC-3' and 5'-GTCA***CTCGAG***AACTCCTTGCTCTGCTGCGTC-3' were used to amplify *nlpD(1-189)* from pMT20. The resulting PCR product was purified, digested with XbaI and XhoI and ligated with similarly digested pNP18.

pMT102

The plasmid pMT102 [*att*HK022 *tetA tetR lacI* P_{lac}::*nlpD*$^{(1-189)}$] was constructed as follows. The primers 5'-TTAGGCACCCCAGGCTTTACAC-3' and 5'-GTCA***AAGCTT***AAACTCCTTGCTCTGCTGCGTC-3' were used to amplify *nlpD(1-189)* from pMT20. The resulting PCR product was purified, digested with XbaI and HindIII and ligated with similarly digested pNP20.

pMT103

The plasmid pMT103 [*att*HK022 *tetA tetR lacI* P_{lac}::*nlpD*$^{(1-115)}$-*mCherry*] was constructed as follows. The primers 5'-TTAGGCACCCCAGGCTTTACAC-3' and 5'-GTCA***CTCGAG***TTTCGGAATGTTCCCATACTGACGG-3' were used to amplify *nlpD(1-115)* from pMT20. The resulting PCR product was purified, digested with XbaI and XhoI and ligated with similarly digested pNP18.

pMT104

The plasmid pMT104 [*att*HK022 *tetA tetR lacI* P_{lac}::*nlpD*$^{(1-115)}$] was constructed as follows. The primers 5’-TTAGGCACCCCAGGCTTTACAC-3’ and 5’-GTCA***AAGCTT***ATTTCGGAATGTTCCCATACTGACGG-3’ were used to amplify *nlpD(1-115)* from pMT20. The resulting PCR product was purified, digested with XbaI and HindIII and ligated with similarly digested pNP20.

pMT105

The plasmid pMT105 [*att*HK022 *tetA tetR lacI* P_{lac}::^{ss}*dsbA-nlpD*$^{(102-175)}$] was constructed as follows. The primers 5’-GTCA***GGATCC***GGACGCATCGTCTATAACCGTCAGTATG-3’ and 5’-GTCA***AAGCTT***CCGCCGATTTATCGCTGC-3’ were used to amplify *nlpD(102-175)* from genomic DNA. The resulting PCR product was purified, digested with BamHI and HindIII and ligated with similarly digested pNP19 [*att*HK022 *tetA tetR lacI* P_{lac}::^{ss}*dsbA-amiB-sfGFP*] [5].

pMT121

The plasmid pMT121 [*att*HK022 *tetA tetR lacI* P_{lac}::^{ss}*dsbA-nlpD*$^{(27-379)}$] was constructed as follows. The *nlpD* containing XbaI/HindIII fragment of pMT18 [*att*HK022 *bla lacI*^{q} P_{lac}::^{ss}*dsbA-nlpD*$^{(27-379)}$] was used to replace the corresponding fragment of pNP20.

pMT147

The plasmid pMT147 [*att*HK022 *tetA tetR lacI* P_{lac}::^{ss}*dsbA-nlpD*$^{(27-379)}$*-mCherry*] was constructed as follows. The primers 5’-TTAGGCACCCCAGGCTTTACAC-3’ and 5’-

GTCA***CTCGAG***TCGCTGCGGCAAATAACGC-3' were used to amplify ^{ss}*dsbA-nlpD(27-379)* from pMT121. The resulting PCR product was purified, digested with XbaI and XhoI and ligated with similarly digested pNP20.

pMT149

The plasmid pMT149 [*att*HK022 *tetA tetR lacI* P_{lac}*::nlpD*$^{(1-30)}$*-mCherry*] was constructed as follows. The primers 5'-TTAGGCACCCCAGGCTTTACAC-3' and 5'-GTCA***CTCGAG***TGAAGTGTCAGAACAGCCTGCC-3' were used to amplify *nlpD(1-30)* from pMT20. The resulting PCR product was purified, digested with XbaI and XhoI and ligated with similarly digested pNP20.

pMT178

The plasmid pMT178 [*att*HK022 *tetA tetR lacI* P_{lac}*::* ^{ss}*dsbA-nlpD*$^{(102-175)}$*-mCherry*] was constructed as follows. The primers 5'-TTAGGCACCCCAGGCTTTACAC-3' and 5'-GCTA***CTCGAG***GCCAGTGATTGGCGTACCG-3' were used to amplify ^{ss}*dsbA-nlpD(102-175)* from pMT105. The resulting PCR product was purified, digested with XbaI and XhoI and ligated with similarly digested pNP20.

pMT179

The plasmid pMT179 [*att*HK022 *tetA tetR lacI* P_{lac}*::* ^{ss}*dsbA-nlpD*$^{(250-379)}$] was constructed as follows. The primers 5'-GTCA***GGATCC***AGTACATCAACCAGTACGCCTATCTCC-3' and 5'-GACGAAAGTGATTGCGCCTACC-3' were used to amplify *nlpD(250-379)* from

pMT20. The resulting PCR product was purified, digested with BamHI and HindIII and ligated with similarly digested pNP19.

pMT180

The plasmid pMT180 [*att*HK022 *tetA tetR lacI* P_{lac}:: ^{ss}*dsbA-nlpD*$^{(250-379)}$*-mCherry*] was constructed as follows. The primers 5’-TTAGGCACCCCAGGCTTTACAC-3’ and 5’-GTCA***CTCGAG***TCGCTGCGGCAAATAACGC-3’ were used to amplify *nlpD(250-379)* from pMT179. The resulting PCR product was purified, digested with XbaI and XhoI and ligated with similarly digested pNP20.

pMT181

The plasmid pMT181 [*att*HK022 *tetA tetR lacI* P_{lac}:: ^{ss}*dsbA-nlpD*$^{(189-379)}$] was constructed as follows. The primers 5’-GTCA***GGATCC***GTTGTGATCAAGCCTGCACAAAATTCC-3’ and 5’-GACGAAAGTGATTGCGCCTACC-3’ were used to amplify *nlpD(189-379)* from pMT20. The resulting PCR product was purified, digested with BamHI and HindIII and ligated with similarly digested pNP19.

pMT182

The plasmid pMT182 [*att*HK022 *tetA tetR lacI* P_{lac}:: ^{ss}*dsbA-nlpD*$^{(189-379)}$*-mCherry*] was constructed as follows. The primers 5’-TTAGGCACCCCAGGCTTTACAC-3’ and 5’-GTCA***CTCGAG***AACTCCTTGCTCTGCTGCGTC-3’ were used to amplify *nlpD(189-379)* from pMT181. The resulting PCR product was purified, digested with XbaI and XhoI and ligated with similarly digested pNP20.

pMT187

This plasmid pMT187 [*aadA repA(ts) lacI*^{q} P$_{lac}$*::envC-LE* SceI *cI857* P$_{\lambda R}$::i-sceI] is a derivative of pBL200 [*aadA repA(ts)* P$_{syn135}$*::ftsN* SceI *cI857* P$_{\lambda R}$::i-sceI] [14] in which the P$_{syn135}$*::ftsN* containing EcoRI/HindIII fragment was replaced with *lacI*^{q} P$_{lac}$*::envC-LE*.

pMT196

The plasmid pMT196 [*attλ cat lacI* P$_{lac}$*::yraP*$^{(1-191)}$] was constructed as follows. The primers 5'-GCTA***TCTAGA***TTAAGAAGGAGATATACATATGAAGGCATTATCGCCAATCGC-3' and 5'-GCTA***AAGCTT***GGCTGCTATTTAATAAACGTAAACGCCG-3' were used to amplify *yraP(1-191)* from genomic DNA. The resulting PCR product was purified, digested with XbaI and HindIII and ligated with similarly digested pHC514 [*attλ cat lacI* P$_{lac}$*::slmA*].

pMT197

The plasmid pMT197 [*attλ cat lacI* P$_{lac}$*::yraP*$^{(1-191)}$*-mCherry*] was constructed as follows. The primers 5'-GCTA***TCTAGA***TTAAGAAGGAGATATACATATGAAGGCATTATCGCCAATCGC-3' and 5'-GCTA***CTCGAG***TTTAATAAACGTAAACGCCGTAGTTACCCG-3' were used to amplify *yraP(1-191)* from genomic DNA. The resulting PCR product was purified, digested with XbaI and XhoI and ligated with a similarly digested pHC514 derivative containing mCherry.

pMT198

The plasmid pMT198 [*attλ cat lacI* P*lac*:: *yraP*$^{(VA\text{-}20,21\text{-}DE)}$] was constructed as follows. The primers 5'-GCTA***TCTAGA***TTAAGAAGGAGATATACATATGAAGGCATTATCGCCAATCGC-3'/5'-CTACGGCAGCCTCATCACAACCTTGCAACAGCAGCG-3' and 5'-GCAAGGTTGTGATGAGGCTGCCGTAGTGGGTACCGC-3'/5'-GCTA***AAGCTT***GGCTGCTATTTAATAAACGTAAACGCCG-3' were used in overlap extension PCR to amplify the *yraP* gene from genomic DNA and to perform site directed mutagenesis to create the (VA-20,21-DE) mutations in the *yraP* gene. The resulting PCR product was purified, digested with XbaI and HindIII and ligated with similarly digested pHC514 [*attλ cat lacI* P*lac*::*slmA*].

pMT199

The plasmid pMT199 [*attλ cat lacI* P*lac*:: *yraP*$^{(VA\text{-}20,21\text{-}DE)}$*-mCherry*] was constructed as follows. The primers 5'-GCTA***TCTAGA***TTAAGAAGGAGATATACATATGAAGGCATTATCGCCAATCGC-3'/5'-CTACGGCAGCCTCATCACAACCTTGCAACAGCAGCG-3' and 5'-GCAAGGTTGTGATGAGGCTGCCGTAGTGGGTACCGC-3'/5'-GCTA***CTCGAG***TTTAATAAACGTAAACGCCGTAGTTACCCG-3' were used in overlap extension PCR to amplify the *yraP* gene from genomic DNA and to perform site directed mutagenesis to create the (VA-20,21-DE) mutations in the *yraP* gene. The resulting PCR product was purified, digested with XbaI and XhoI and ligated with a similarly digested pHC514 derivative containing mCherry.

pMT209

The plasmid pMT209 [*attλ cat lacI* P_{lac}:: ^{ss}*dsbA-yraP*$^{(24-191)}$] was constructed in two steps as follows. The primers 5'-GCTA***GGATCC***GTAGTGGGTACCGCTGCTG-3' and 5'-GCTA***AAGCTT***GGCTGCTATTTAATAAACGTAAACGCCG-3' were used to amplify *yraP(24-191)* from genomic DNA. The resulting PCR product was purified, digested with BamHI and HindIII and ligated with similarly digested pNP19 to make pMT200 [*att*HK022 *tetA tetR lacI* P_{lac}:: ^{ss}*dsbA-yraP*$^{(24-191)}$]. The *yraP* containing XbaI/HindIII fragment of pMT200 was then used to replace the corresponding fragment of pHC514.

pMT210

The plasmid pMT210 [*attλ cat lacI* P_{lac}:: ^{ss}*dsbA-yraP*$^{(24-191)}$*-mCherry*] was constructed as follows. The primers 5'-TTAGGCACCCCAGGCTTTACAC-3' and 5'-GCTA***CTCGAG***TTTAATAAACGTAAACGCCGTAGTTACCCG-3' were used to amplify ^{ss}*dsbA-yraP(24-191)* from pMT200. The resulting PCR product was purified, digested with XbaI and XhoI and ligated with a similarly digested pHC514 derivative containing mCherry.

pMT224

The plasmid pMT224 [*bla araC* P_{ara}::*sulA*] was constructed as follows. The *sulA* containing XbaI/HindIII fragment of pMT74 [*tetA tetR lacI*^{q} P_{lac}::*sulA*] was used to replace the corresponding fragment of pBAD24 [*bla araC* P_{ara}::*empty*] [6].

REFERENCES

1. Guyer MS, Reed RR, Steitz JA, Low KB. Identification of a sex-factor-affinity site in E. coli as gamma delta. Cold Spring Harb Symp Quant Biol. 1981;45 Pt 1:135–40.

2. Johnson JE, Lackner LL, Hale CA, de Boer PAJ. ZipA is required for targeting of DMinC/DicB, but not DMinC/MinD, complexes to septal ring assemblies in Escherichia coli. J Bacteriol. 2004 Apr 1;186(8):2418–29.

3. Bernhardt TG, de Boer PAJ. Screening for synthetic lethal mutants in Escherichia coli and identification of EnvC (YibP) as a periplasmic septal ring factor with murein hydrolase activity. Mol Microbiol. 2004 Jun;52(5):1255–69.

4. Uehara T, Dinh T, Bernhardt TG. LytM-domain factors are required for daughter cell separation and rapid ampicillin-induced lysis in Escherichia coli. J Bacteriol. 2009 Aug;191(16):5094–107.

5. Peters NT, Dinh T, Bernhardt TG. A fail-safe mechanism in the septal ring assembly pathway generated by the sequential recruitment of cell separation amidases and their activators. J Bacteriol. 2011 Sep;193(18):4973–83.

6. Guzman LM, Belin D, Carson MJ, Beckwith J. Tight regulation, modulation, and high-level expression by vectors containing the arabinose PBAD promoter. J Bacteriol. 1995 Jul 1;177(14):4121–30.

7. Haldimann A, Wanner BL. Conditional-replication, integration, excision, and retrieval plasmid-host systems for gene structure-function studies of bacteria. J Bacteriol. 2001 Nov 1;183(21):6384–93.

8. Datsenko KA, Wanner BL. One-step inactivation of chromosomal genes in Escherichia coli K-12 using PCR products. Proc Natl Acad Sci USA. 2000 Jun 6;97(12):6640–5.

9. Tsang M-J, Bernhardt TG. A role for the FtsQLB complex in cytokinetic ring activation revealed by an ftsL allele that accelerates division. Mol Microbiol. 2015 Mar;95(6):925–44.

10. Bernhardt TG, de Boer PAJ. SlmA, a nucleoid-associated, FtsZ binding protein required for blocking septal ring assembly over Chromosomes in E. coli. Mol Cell. 2005 May 27;18(5):555–64.

11. Uehara T, Parzych KR, Dinh T, Bernhardt TG. Daughter cell separation is controlled by cytokinetic ring-activated cell wall hydrolysis. EMBO J. 2010 Apr 21;29(8):1412–22.

12. Cho H, McManus HR, Dove SL, Bernhardt TG. Nucleoid occlusion factor SlmA is a DNA-activated FtsZ polymerization antagonist. Proc Natl Acad Sci USA. 2011 Mar 1;108(9):3773–8.

13. Yang DC, Tan K, Joachimiak A, Bernhardt TG. A conformational switch controls cell wall-remodelling enzymes required for bacterial cell division. Mol Microbiol. 2012 Aug;85(4):768–81.

14. Liu B, Persons L, Lee L, de Boer PAJ. Roles for both FtsA and the FtsBLQ subcomplex in FtsN-stimulated cell constriction in Escherichia coli. Mol Microbiol. 2015 Mar;95(6):945–70.
